# Ferritinophagy is required for the induction of ferroptosis by the bromodomain protein BRD4 inhibitor (+)-JQ1 in cancer cells

**DOI:** 10.1038/s41419-019-1564-7

**Published:** 2019-04-15

**Authors:** Shiyao Sui, Jian Zhang, Shouping Xu, Qin Wang, Peiyuan Wang, Da Pang

**Affiliations:** 1 Department of Breast Surgery, Harbin Medical University Cancer Hospital, 150 Haping Road, 150081 Harbin, China; 2 Heilongjiang Academy of Medical Sciences, 157 Baojian Road, 150086 Harbin, China

## Abstract

(+)-JQ1 is an inhibitor of the tumor-driver bromodomain protein BRD4 and produces satisfactory effects because it efficiently increases apoptosis. Ferroptosis is an oxidative cell death program differing from apoptosis. Ferroptosis is characterized by high levels of iron and reactive oxygen species and has been confirmed to suppress tumor growth. In this study, BRD4 expression in cancer and its influence on the prognosis of cancer patients were analyzed using data from public databases. In addition, the effect of the BRD4 inhibitor (+)-JQ1 on ferroptosis was investigated via a series of in vitro assays. A nude mouse model was used to evaluate the function of (+)-JQ1 in ferroptosis in vivo. The potential mechanisms by which (+)-JQ1 regulates ferroptosis were explored. The results showed that BRD4 expression levels were higher in cancer tissues than in normal tissues and were related to poor prognosis in cancer patients. Furthermore, ferroptosis was induced under (+)-JQ1 treatment and BRD4 knockdown, indicating that (+)-JQ1 induces ferroptosis via BRD4 inhibition. Moreover, the anticancer effect of (+)-JQ1 was enhanced by ferroptosis inducers. Further studies confirmed that (+)-JQ1 induced ferroptosis via ferritinophagy, which featured autophagy enhancement by (+)-JQ1 and increased iron levels. Subsequently, the reactive oxygen species levels were increased by iron via the Fenton reaction, leading to ferroptosis. In addition, expression of the ferroptosis-associated genes *GPX4*, *SLC7A11*, and *SLC3A2* was downregulated under (+)-JQ1 treatment and BRD4 knockdown, indicating that (+)-JQ1 may regulate ferroptosis by controlling the expression of ferroptosis-associated genes regulated by BRD4. Finally, (+)-JQ1 regulated ferritinophagy and the expression of ferroptosis-associated genes via epigenetic inhibition of BRD4 by suppressing the expression of the histone methyltransferase G9a or enhancing the expression of the histone deacetylase SIRT1. In summary, the BRD4 inhibitor (+)-JQ1 induces ferroptosis via ferritinophagy or the regulation of ferroptosis-associated genes through epigenetic repression of BRD4.

## Introduction

The bromodomain and extraterminal domain (BET) family of proteins comprises BRD2, BRD3, BRD4 and BRDt^1^. The bromodomain structure consists of four alpha helices separated by variable loop regions, which can recognize acetylation sites and recruit transcription factors^2^. Based on its strong effect on transcriptional regulation, the role of the BET family in the promotion of biological behavior of cancer cells has been identified^3^. Furthermore, the BRD4 inhibitor (+)-JQ1 (JQ1) has been shown to suppress the proliferation of cancer cells^4,5^, indicating that JQ1 may be a new therapeutic agent for cancer treatment. However, the clinical application of JQ1 is limited. Since some cancer cells are insensitive to apoptosis, cancer cells remain after JQ1 treatment, subsequently leading to treatment failure^6,7^. Therefore, new drugs or models need to be identified to overcome the obstacles associated with JQ1 treatment.

Ferroptosis is a newly discovered type of cell death that is characterized by high intracellular levels of iron and reactive oxygen species (ROS)^8,9^. Ferroptosis is mainly caused by deficits in the production of reduced glutathione or by the dysfunction of glutathione peroxidase 4 (GPX4), which are ROS eliminators^10^. Excess levels of ROS induce lipid peroxidation and lead to the disintegration of lipid membranes, followed by cell death^11,12^. Ferroptosis inducers, including 1S,3R-RSL3 (RSL3), which inhibits the function of GPX4, and erastin, which decreases the level of reduced glutathione via the inhibition of system x_c_^−^, have been confirmed to exhibit anticancer effects^13,14^. In addition, ferroptosis strengthened the anticancer effect of the apoptosis-inducer cisplatin in head and neck cancer cells^15^, indicating that ferroptosis inducers could be used to enhance the effect of traditional anticancer drugs. However, whether ferroptosis plays a role in the anticancer effect of JQ1 is unknown.

In this study, we explored the relationship between JQ1 and ferroptosis. We used public databases to investigate BRD4 expression in cancer tissues and its association with the prognosis of cancer patients. We discovered that BRD4 expression was higher in cancer tissues than in normal tissues and was associated with poorer prognoses in cancer patients, indicating that targeting BRD4 may confer a clinical benefit in cancer patients. Furthermore, ferroptosis played a role in the anticancer effect of JQ1 both in vitro and in vivo, and the ferroptosis inducers RSL3, erastin, and sorafenib enhanced the anticancer effect of JQ1. Moreover, JQ1 enhanced ferroptosis via the increase in ferritinophagy or the regulation of ferroptosis-associated genes through BRD4 inhibition. Finally, we found that JQ1 regulated ferritinophagy and ferroptosis-associated genes via epigenetic inhibition of BRD4 by suppressing the expression of the histone methyltransferase G9a or enhancing the expression of the histone deacetylase SIRT1.

## Results

### BRD4 expression is upregulated in multiple types of cancer

**Fig. 1:**
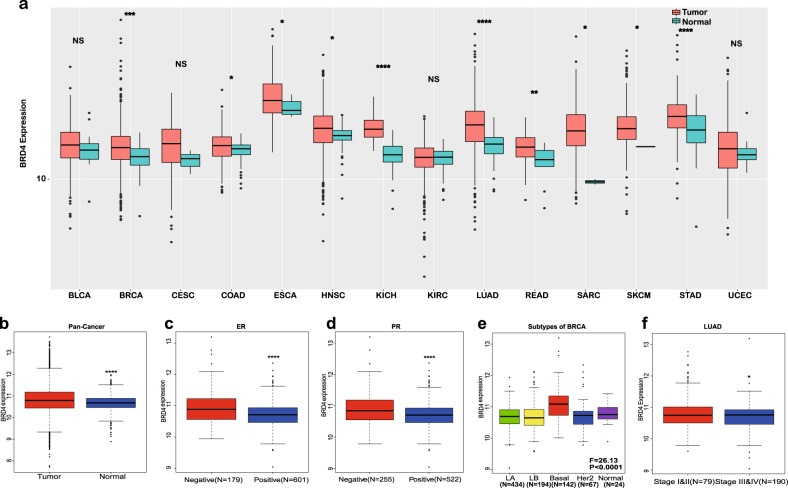
Expression of BRD4 in cancer patients included in The Cancer Genome Atlas (TCGA) database. **a** BRD4 expression was higher in cancer tissues (BRCA, COAD, ESCA, HNSC, KICH, LUAD, STAD, READ, SARC, SKCM, BLCA, CESC, KIRC, and UCEC) than in tissues of healthy subjects included in the TCGA database. **b** BRD4 expression was higher in pan-cancer patients (*N* = 8031) than in healthy subjects (*N* = 722) included in the TCGA database. **c**, **d** BRD4 expression was higher in estrogen receptor (ER)-negative (**c**) (*N* = 179)/progesterone receptor (PR)-negative (**d**) (*N* = 255) BRCA patients than in ER-positive (*N* = 601)/PR-positive (*N* = 522) BRCA patients. **e** Expression of BRD4 in different subtypes of BRCA patients. **f** BRD4 expression was higher in Tumor, Node, Metastasis (TNM) stage III and IV LUAD patients (*N* = 190) than in TNM stage I and II LUAD patients (*N* = 79) (*P < 0.05, **P < 0.01, ***P < 0.001, ****P < 0.0001, NS no significance)

Since the anticancer effect of JQ1 is mainly derived from its inhibition of BRD4, we first explored the role of BRD4 in cancer. The expression of BRD4 in tissues of patients with various types of cancer was investigated in data from The Cancer Genome Atlas (TCGA). Specific data, including the number of patients and healthy subjects and the *P* values, are listed in Table 1. We discovered that BRD4 expression was higher in tissues from patients with breast cancer (BRCA), colon adenocarcinoma (COAD), esophageal cancer (ESCA), head and neck squamous cell carcinoma (HNSC), kidney chromophobe (KICH), lung squamous cell carcinoma (LUAD), rectal adenocarcinoma (READ), stomach adenocarcinoma (STAD), sarcoma (SARC), and skin cutaneous melanoma (SKCM) than in tissues from healthy subjects (Fig. 1a). However, for bladder cancer (BLCA), cervical squamous cell carcinoma (CESC), kidney renal clear cell carcinoma (KIRC), and uterine corpus endometrial carcinoma (UCEC), the expression of BRD4 in tissues from patients was not significantly higher than that in tissues from healthy subjects (Fig. 1a). Similarly, the expression of BRD4 was higher in a total analysis of pan-cancer patients (Fig. 1b). Furthermore, we evaluated different subtypes of individual cancers such as BRCA and LUAD. In BRCA, higher BRD4 expression was found in estrogen receptor (ER)-negative and progesterone receptor (PR)-negative cohorts that indicate worse prognosis (Fig. 1c, d). Moreover, BRD4 expression was higher in basal-like BRCA, which has the lowest survival rate (Fig. 1e). Lastly, among different stages of LUAD, later Tumor, Node, Metastasis (TNM) stages exhibited higher BRD4 expression (BRD4 expression was higher in TNM stages III and IV than in TNM stages I and II (Fig. 1f). In summary, BRD4 expression is higher in cancer tissues than in normal tissues and is positively associated with more severe subtypes and stages.

**Table 1:** Number of samples and corresponding significance of BRD4 expression

Type of cancer	Number of patients	Number of healthy subjects	Significance
BRCA	1104	114	****
COAD	287	41	*
ESCA	185	11	*
HNSC	522	44	*
KICH	66	25	****
LUAD	502	51	****
READ	94	10	**
SARC	260	2	*
SKCM	472	1	*
STAD	415	35	****
BLCA	407	19	ns
CESC	305	3	ns
KIRC	533	72	ns
UCEC	176	24	ns
Pan-Cancer	8031	722	****

*p < 0.05; **p < 0.01; ***p < 0.001; ****p < 0.0001; ns no significance

### Higher BRD4 expression is correlated with poorer survival outcomes

**Fig. 2:**
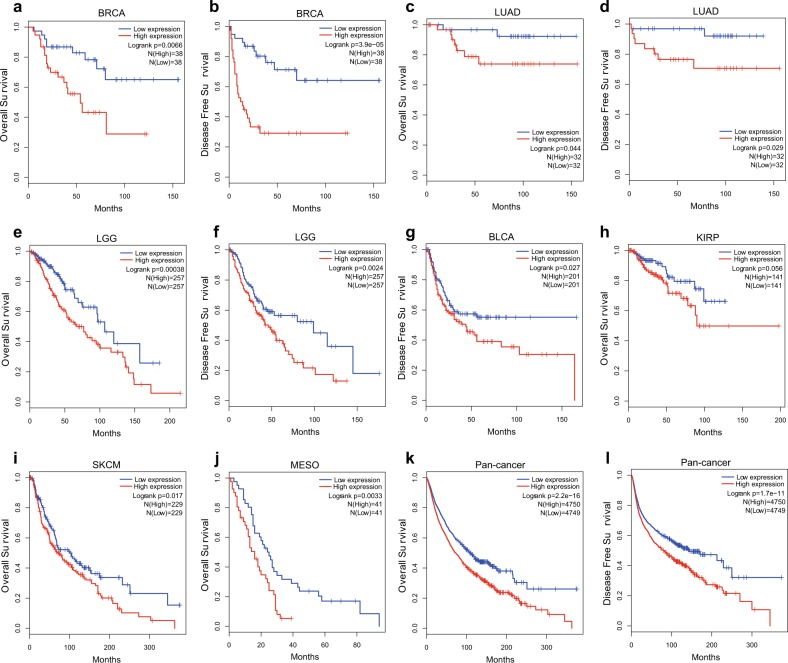
Prognosis of patients is negatively correlated with BRD4 expression. **a**–**f** Higher BRD4 expression was correlated with both poorer overall survival (OS) and poorer disease-free survival (DFS) in BRCA (**a**, **b**) (High/Low: *N* = 38), LUAD (**c**, **d**) (High/Low: *N* = 32), and LGG (**e**, **f**) (High/Low: *N* = 257) patients in The Cancer Genome Atlas (TCGA) database. **g**–**j** Higher BRD4 expression was correlated with poorer DFS in BLCA (**g**) patients (High/Low: *N* = 201) and with poorer OS in KIRP (**h**) (High/Low: *N* = 141), SKCM (**i**) (High/Low: *N* = 229), and MESO (**j**) (High/Low: *N* = 41) patients in the TCGA database. **k**, **l** Higher BRD4 expression was correlated with both poorer OS (**k**) and poorer DFS (**l**) in pan-cancer patients (High: *N* = 4750, Low: *N* = 4749) in the TCGA database

Next, we analyzed the relationship between BRD4 expression and prognosis in cancer patients included in the TCGA database. We found that in BRCA (Fig. 2a, b), LUAD (Fig. 2c, d), and lower-grade glioma (LGG) (Fig. 2e, f), BRD4 expression was positively correlated with both poor overall survival (OS) and disease-free survival (DFS). However, in BLCA (Fig. 2g), BRD4 expression was positively correlated only with poor DFS. In kidney renal papillary cell carcinoma (KIRP) (Fig. 2h), SKCM (Fig. 2i), and mesothelioma (MESO) (Fig. 2j), increased BRD4 expression was positively correlated only with poor OS, although in KIRP, no significant correlation with OS was found. Furthermore, in a total analysis of pan-cancer patients, BRD4 expression was positively correlated with poor OS (Fig. 2k) and DFS (Fig. 2l). In summary, BRD4 expression is negatively related to the survival of cancer patients, and targeting BRD4 may confer a clinical benefit in cancer patients.

### JQ1 induces ferroptosis in BRCA and LUAD cells

**Fig. 3:**
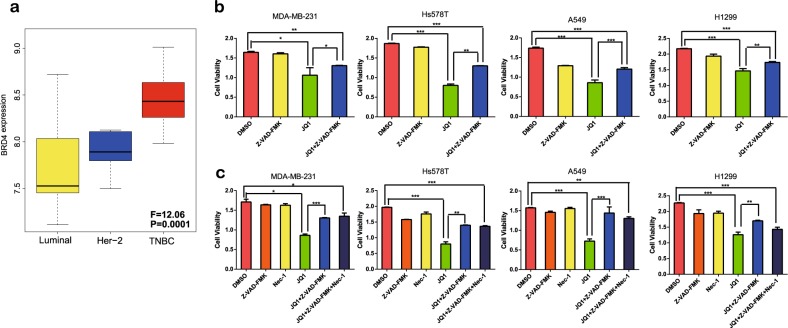
Exploration of cell death induced by JQ1 in vitro. **a** Expression of BRD4 in different types of BRCA cell lines from the Cancer Cell Line Encyclopedia database. **b** The apoptosis inhibitor Z-VAD-FMK (10 μM) did not fully reverse the anticancer effect of JQ1 (10 μM) in the MDA-MB-231 and Hs578T BRCA cell lines or the A549 and H1299 LUAD cell lines. **c** A necrosis inhibitor nec-1 (20 μM) did not influence the viability of cells treated with JQ1 (10 μM) plus Z-VAD-FMK (10 μM) in the MDA-MB-231 and Hs578T BRCA cell lines or the A549 and H1299 LUAD cell lines. An equal volume of dimethyl sulfoxide was used as the control; cell viability was assessed after the application of the drugs for 24 h. *P < 0.05, **P < 0.01, ***P < 0.001. The data represent the results of at least three independent experiments

Next we explored the anticancer effect of JQ1, since it can efficiently suppress BRD4 expression^16^. First, we investigated the expression of BRD4 in different subtypes of BRCA cell lines using the Cancer Cell Line Encyclopedia (CCLE) database (Fig. 3a) and found that the expression of BRD4 is higher in triple-negative breast cancer (TNBC) cell lines than in cell lines of the luminal and HER-2 subtypes. Therefore, we selected the MDA-MB-231 and Hs578T TNBC cell lines for subsequent experiments. Furthermore, as described above, BRD4 expression is increased in higher TNM stages of LUAD, thus the LUAD cell lines A549 and H1299 were included in the experiments.

Since JQ1 has been reported to induce apoptosis^17^, we applied Z-VAD-FMK, a pan-caspase inhibitor that can suppress apoptosis to explore the type of death induced by JQ1. Surprisingly, treatment with Z-VAD-FMK could not fully inhibit the death induced by JQ1 (Fig. 3b). Then we investigated whether necrosis participates in JQ1-induced cell death and found that necrosis was not involved in JQ1-induced death, as the necrosis inhibitor necrostatin-1 (nec-1) did not influence the viability of cells treated with JQ1 plus Z-VAD-FMK (Fig. 3c). This result indicates that cell death programs other than apoptosis and necrosis are induced by JQ1.

**Fig. 4:**
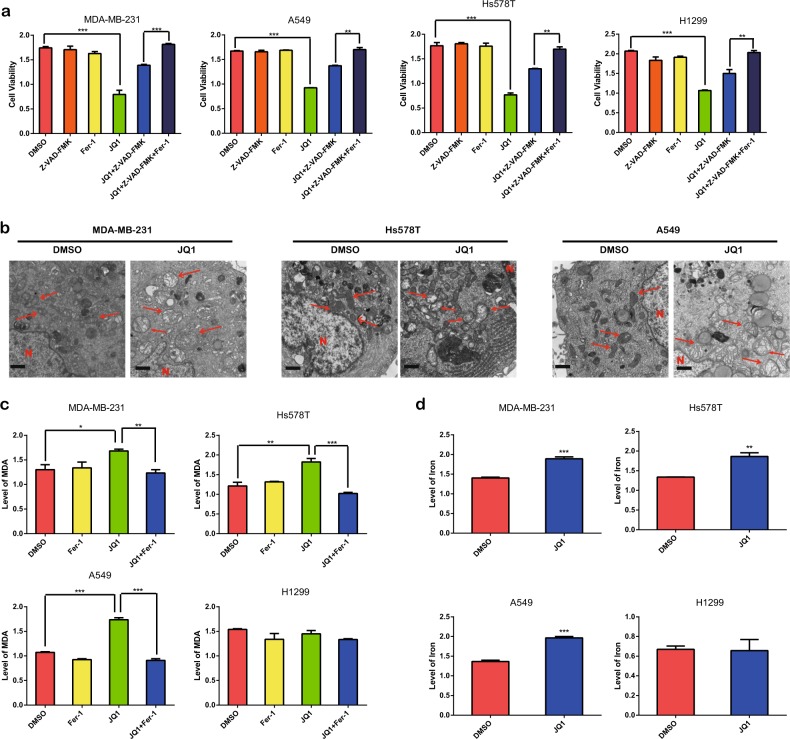
JQ1 induces ferroptosis in vitro. **a** In combination with Z-VAD-FMK (10 μM), the ferroptosis inhibitor fer-1 (1 μM) inhibited the anticancer effect of JQ1 (10 μM) to a higher degree in the MDA-MB-231 and Hs578T BRCA cell lines and the A549 and H1299 LUAD cell lines. **b** Representative electron microscopic images and quantification of swollen mitochondria in the dimethyl sulfoxide (DMSO) and JQ1 groups. Scale bar = 500 nm. The arrows denote mitochondria, and the nucleus is denoted by the letter N. **c**, **d** The levels of reactive oxygen species (ROS) (**c**) and iron (**d**) were increased with JQ1 (10 μM) treatment in the MDA-MB-231 and Hs578T BRCA cell lines and the A549 LUAD cell line but not in the H1299 cell line. An equal volume of DMSO was used as the control; cell viability and levels of iron and ROS were assessed after the application of the drugs for 24 h. *P < 0.05, **P < 0.01, ***P < 0.001. The data represent the results of at least three independent experiments

As ferroptosis is a newly discovered type of cell death and has been identified in cancer cells^18,19^, we investigated whether ferroptosis is involved in JQ1-induced cell death. Surprisingly, in cells treated with the combination of Z-VAD-FMK and the inhibitor of ferroptosis, ferrostatin-1 (fer-1)^8,20^, JQ1-induced cell death was inhibited more noticeably than in cells treated with JQ1 plus Z-VAD-FMK (Fig. 4a). Furthermore, we performed ultrastructural analysis via transmission electron microscopy and observed more swollen mitochondria in the JQ1-treated groups, indicating an increase in intracellular ROS levels with JQ1 treatment (Fig. 4b). Then we investigated the two hallmarks of ferroptosis, the levels of iron and ROS in cells treated with JQ1. Our studies confirmed that the level of malondialdehyde (MDA), which is the final product of lipid peroxidation induced by ROS^21^, was appreciably increased in cancer cell lines treated with JQ1 and was inhibited by treatment with fer-1 (Fig. 4c). In addition, the level of iron was increased in cells treated with JQ1 (Fig. 4d). However, alterations in the ROS and iron levels were not observed in H1299 cells. In summary, ferroptosis is involved in the anticancer effect of JQ1. Furthermore, cell viability was also decreased by knockdown of BRD4, and this decrease was reversed by fer-1 treatment (Fig. S1c). In addition, the levels of iron (Fig. S1d) and ROS (Fig. S1e) were increased by BRD4 knockdown (Fig. S1a). Moreover, we performed invasion and migration assays and found that fer-1 treatment enhanced the invasion (Fig. S3a, c) and migration (Fig. S3b, d) abilities. However, considering that fer-1 inhibits ferroptosis, the enhanced invasion and migration may derive from the increase in cell viability mediated by fer-1. In sum, we can infer that JQ1 induces ferroptosis in vitro through the suppression of BRD4 expression.

### Ferroptosis is involved in the anticancer effect of JQ1 in vivo

**Fig. 5:**
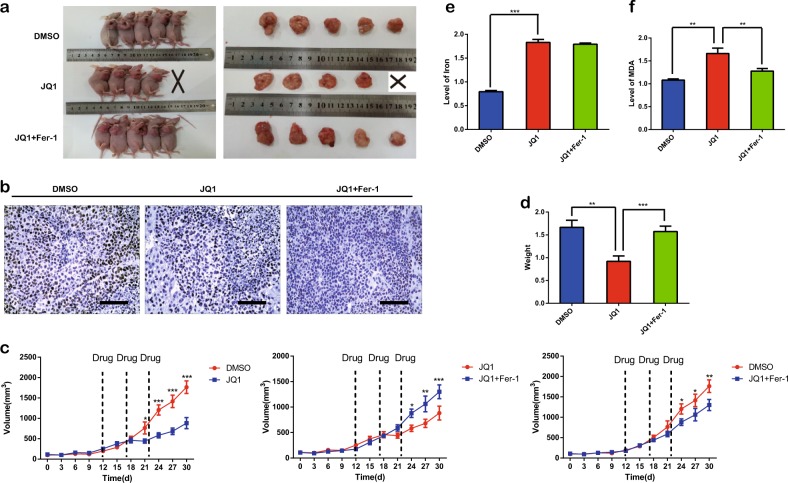
The anticancer effect of JQ1 is suppressed by fer-1 in vivo. **a** Tumors in the JQ1 (50 mg/kg) group were smaller than those in the dimethyl sulfoxide (DMSO) group and the JQ1 (50 mg/kg) plus fer-1 (1 mg/kg) group. **b** The immunohistochemical results showed that BRD4 expression in tumors in the JQ1 group and the JQ1 plus fer-1 group was lower than that in tumors in the DMSO group. **c** Tumor sample volumes are shown using a time-course line graph. **d** The weight of tumors in the JQ1 group was lower than that of tumors in the DMSO group and the JQ1 plus fer-1 group. **e** The level of iron was increased with JQ1 treatment but was not influenced by fer-1 treatment. **f** The level of reactive oxygen species was increased with JQ1 treatment and was decreased with fer-1 treatment. The data are presented as the means ± SDs. An equal volume of DMSO was used as the control. Scale bar = 50 μm. *P < 0.05, **P < 0.01, ***P < 0.001

To further confirm the effect of JQ1 in ferroptosis, we established nude mouse xenograft models. One mouse from the JQ1 group died 1 day before the tumors were harvested and was discarded. Treatment with JQ1 plus fer-1 efficiently reversed the anticancer effect of JQ1 in the xenograft models (Fig. 5a, c). Moreover, the weight of the tumors from the JQ1 group was lower than that of the tumors from the control and JQ1 plus fer-1 groups (Fig. 5d). Immunohistochemistry showed that BRD4 expression in the JQ1 and JQ1 plus fer-1 groups was lower than that in the dimethyl sulfoxide (DMSO) group (Fig. 5b). In addition, we assessed the levels of iron and ROS in these tumors. As expected, the JQ1 group showed higher levels of iron (Fig. 5e) than the DMSO group and higher levels of ROS (Fig. 5f) than the DMSO and JQ1 plus fer-1 groups. Overall, we can infer that JQ1 decreases tumor growth via ferroptosis mediated by BRD4 suppression in vivo.

### Anticancer effect of JQ1 is enhanced by the ferroptosis-inducer RSL3

**Fig. 6:**
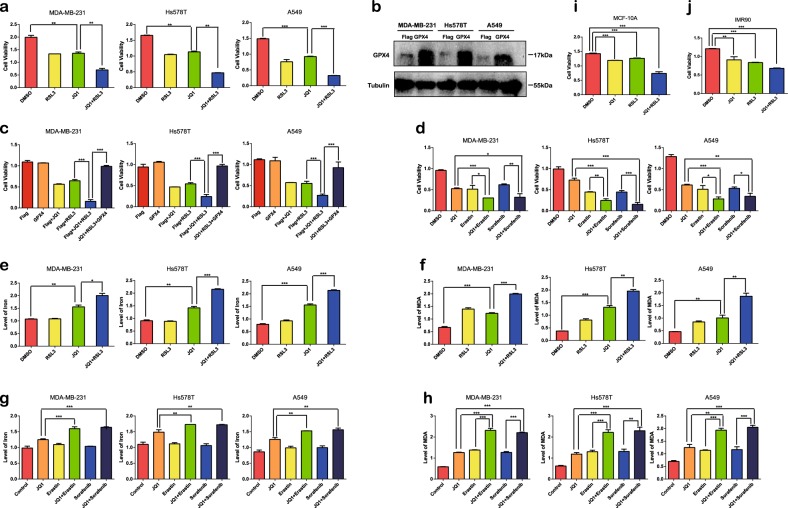
The anticancer effect of JQ1 is enhanced by ferroptosis inducers in vitro. **a** The anticancer effect of JQ1 (10 μM) was enhanced by the ferroptosis-inducer RSL3 (1 μM) in the MDA-MB-231 and Hs578T BRCA cell lines and in the A549 LUAD cell line. **b**, **c** Anticancer effect of JQ1 (10 μM) and RSL3 was abolished in GPX4-upregulated cancer cells. **d** The anticancer effect of JQ1 (10 μM) was enhanced by the ferroptosis-inducer erastin (20 μM) and sorafenib (10 μM) in the MDA-MB-231 and Hs578T BRCA cell lines and in the A549 LUAD cell line. **e**, **f** The levels of iron (**e**) and reactive oxygen species (ROS) (**f**) were increased in cells treated with JQ1 (10 μM) plus RSL3 (1 μM) compared with that in cells treated with either agent alone. **g**, **h** The levels of iron (**g**) and ROS (**h**) were increased in cells treated with JQ1 (10 μM) plus erastin (20 μM) or sorafenib (10 μM) compared with that in cells treated with either agent alone. **i**, **j** Treatment with JQ1 (10 μM) plus RSL3 (1 μM) or either agent alone deceased viability in MCF-10A nontumorigenic breast epithelial cells (**i**) and IMR90 lung fibroblasts (**j**). An equal volume of dimethyl sulfoxide was used as the control; cell viability and levels of iron and ROS were assessed after the application of the drugs for 24 h. *P < 0.05, **P < 0.01, ***P < 0.001. The data represent the results of at least three independent experiments

Then we explored whether a ferroptosis inducer could enhance the anticancer ability of JQ1 in cancer cells. We treated cancer cells with RSL3, which induces ferroptosis via the inhibition of GPX4, which is an ROS scavenger. We discovered that the combination of JQ1 with RSL3 induced cell death to a higher degree than the application of either RSL3 or JQ1 alone (Fig. 6a). Further experiments confirmed that the synergistic effect was abolished when GPX4 was overexpressed (Fig. 6b, c). In addition, we explored whether the anticancer effect of JQ1 could be enhanced by other ferroptosis inducers. JQ1 exhibited a synergistic effect with erastin and sorafenib, which are system x_c_^−^ inhibitors (Fig. 6d). Moreover, under treatment with the combination of JQ1 and RSL3, erastin, or sorafenib, the levels of iron (Fig. 6e, g) and ROS (Fig. 6f, h) in cancer cells was increased to a higher degree than the application of JQ1 alone. Furthermore, the invasion (Fig. S3e, g) and migration (Fig. S3f, h) abilities of BRCA and LUAD cells were reduced more appreciably under treatment with JQ1 plus RSL3 than with either agent alone. However, considering that RSL3 can induce ferroptosis, the suppression of invasion and migration may derive from the enhancement of cell death by RSL3. In summary, the anticancer effect of JQ1 in cancer cells could be enhanced by ferroptosis inducers. Moreover, we found that JQ1 or RSL3 treatment alone led to the death of MCF-10A nontumorigenic epithelial breast cells (Fig. 6i) and IMR90 lung fibroblast cells (Fig. 6j), and this effect was enhanced under the combination of JQ1 and RSL3, indicating that ferroptosis also occurs in normal cells.

### JQ1 induces ferroptosis in cancer cells via ferritinophagy

**Fig. 7:**
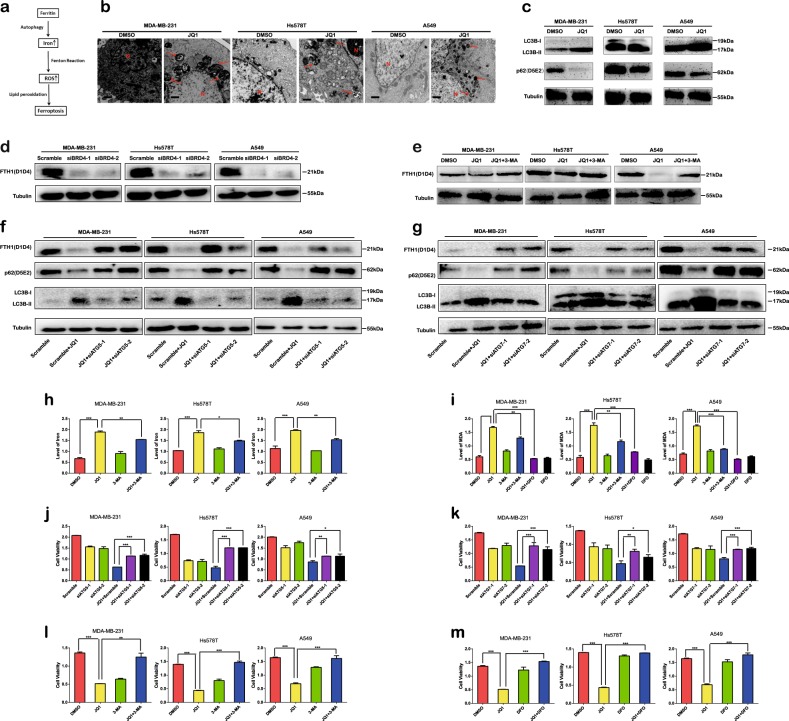
JQ1 regulates ferroptosis via ferritinophagy. **a** Schematic of the ferritinophagy process. **b** Representative electron microscopic images and quantification of autophagosomes in the dimethyl sulfoxide (DMSO) group and the JQ1 group. Scale bar = 500 nm. The arrows denote mitochondria, and the nucleus is denoted by the letter N. **c** Expression of the autophagy markers LC3B-II/LC3B-I was increased and that of SQSTM1 was decreased under treatment with JQ1 (10 μM, 24 h). **d** The expression level of FTH1 was decreased under knockdown of BRD4; ratio of knockdown of BRD4 is shown in Fig. S1b. **e**–**g** The expression level of FTH1 was decreased under treatment with JQ1 (10 μM, 24 h), and this decrease was reversed under treatment with the combination of JQ1 (10 μM, 24 h) and 3-methyladenine (3-MA; 50 μM, 24 h) (**e**) or treatment with the combination of JQ1 (10 μM, 24 h) and knockdown of ATG5 (**f**) or ATG7 (**g**) in the MDA-MB-231 and Hs578T BRCA cell lines and in the A549 LUAD cell line; ratio of ATG5 or ATG7 knockdown is shown in Fig S4a. **h** The level of iron was increased under treatment with JQ1 (10 μM), and this increase was reversed under treatment with the combination of JQ1 (10 μM) and 3-MA (50 μM) in the MDA-MB-231 and Hs578T BRCA cell lines and in the A549 LUAD cell line. **i** The level of reactive oxygen species (ROS) was increased under treatment with JQ1 (10 μM), and this increase was reversed under treatment with the combination of JQ1 (10 μM) and 3-MA (50 μM) or the combination of JQ1 (10 μM) and the iron chelator desferrioxamine (DFO; 100 μM) in the MDA-MB-231 and Hs578T BRCA cell lines and in the A549 LUAD cell line. **j**, **k** Cell viability was decreased under treatment with JQ1 (10 μM), and this decrease was reversed under treatment with the combination of JQ1 (10 μM) and knockdown of ATG5 (**j**) or ATG7 (**k**). **l** Cell viability was decreased under treatment with JQ1 (10 μM), and this decrease was reversed under treatment with the combination of JQ1 (10 μM) and 3-MA (50 μM) in the MDA-MB-231 and Hs578T BRCA cell lines and in the A549 LUAD cell line. **m** Cell viability was decreased under treatment with JQ1 (10 μM), and this decrease was reversed under treatment with the combination of JQ1 (10 μM) and the iron chelator DFO (100 μM) in the MDA-MB-231 and Hs578T BRCA cell lines and in the A549 LUAD cell line. An equal volume of DMSO was used as the control; cell viability and levels of iron and ROS were assessed after the application of the drugs for 24 h. *P < 0.05, **P < 0.01, ***P < 0.001. The data represent the results of at least three independent experiments

Next, we explored the potential mechanism by which JQ1 regulates ferroptosis. Recent reports have confirmed that ferritinophagy is an effective inducer of ferroptosis^22,23^. In this process, ferritin heavy chain 1 (FTH1) is degraded via autophagy, leading to increased intracellular levels of iron. Then the level of ROS is augmented by this excess iron via the Fenton reaction (Fig. 7a)^24^. We found that, under treatment with JQ1, the number of autophagosomes was appreciably increased, as observed via transmission electron microscopy (Fig. 7b). Furthermore, the ratio of LC3B-II/LC3B-I was upregulated and the level of the autophagic cargo SQSTM1/p62 was downregulated under JQ1 treatment (Fig. 7c). Moreover, JQ1 treatment and BRD4 knockdown led to decreased FTH1 expression, indicating that JQ1 induces the degradation of FTH1 via BRD4 inhibition (Fig. 7d, e). In addition, when autophagy was inhibited via knockdown of autophagy-related 5 (ATG5) (Fig. 7f) or autophagy-related 7 (ATG7) (Fig. 7g) or by treatment with 3-methyladenine (3-MA) (Fig. 7e), the JQ1-mediated suppression of FTH1 expression was reversed. Furthermore, in cells treated with JQ1, we observed an increase in the iron and ROS levels that was abolished by treatment with JQ1 plus knockdown of either ATG5 (Fig. S4b, c) or ATG7 (Fig. S4d, e) or by treatment with 3-MA (Fig. 7h, i), indicating the induction of ferritinophagy under JQ1 treatment. Moreover, we found that the increase in ROS levels induced by JQ1 was alleviated by treatment with the combination of JQ1 plus iron chelator desferrioxamine (DFO) (Fig. 7i, yellow and purple columns), indicating that, under JQ1 treatment, the levels of ROS increased via the augmentation of iron levels. Then we investigated whether the ferroptosis induced by JQ1 is initiated via ferritinophagy. The viability of cancer cells treated with the combination of JQ1 and knockdown of ATG5 (Fig. 7j) or ATG7 (Fig. 7k) or the combination of JQ1 and 3-MA (Fig. 7l) or DFO (Fig. 7m) was increased compared to that of cells treated with JQ1 alone; however, treatment with knockdown of ATG5 or ATG7 or treatment with either 3-MA or DFO alone did not enhance cell viability. Therefore, we inferred that JQ1 induces ferroptosis via ferritinophagy in cancer cells.

### JQ1 induces ferroptosis via the regulation of ferroptosis- or autophagy-associated genes

**Fig. 8:**
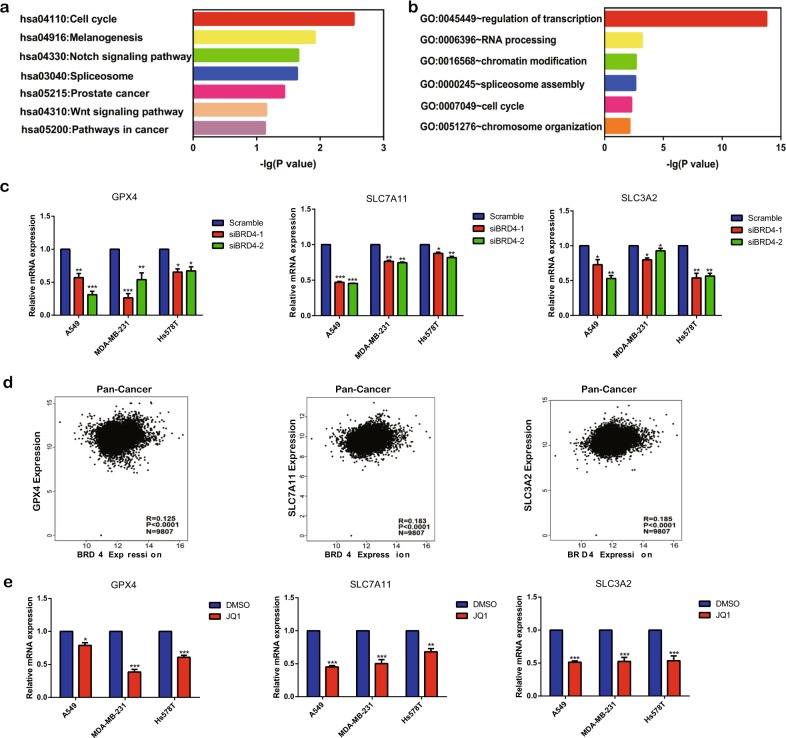
JQ1 induces ferroptosis via the regulation of genes associated with ferroptosis. **a**, **b** Kyoto Encyclopedia of Genes and Genomes (**a**) term enrichment and Gene Ontology (**b**) pathway analysis data were analyzed using the DAVID Functional Annotation Bioinformatics Microarray Analysis tool (https://david.ncifcrf.gov/). The vertical axis represents the biological process or pathway category, and the horizontal axis represents −log_10_ (*P* value) of these significant biological processes or pathways. **c** The expression of the ferroptosis-associated genes *GPX4*, *SLC7A11*, and *SLC3A2* was downregulated with BRD4 knockdown. The gene expression values were normalized to GAPDH expression. The expression values in the Scramble groups were normalized to 1. The data represent the results of at least three independent experiments. **d** The expression of BRD4 was positively related to the expression of *GPX4* (correlation coefficient (*R*) = 0.125), *SLC7A11* (*R* = 0.183), and *SLC3A2* (*R* = 0.185). The data were analyzed through the calculation of pairwise Pearson correlation coefficients. **e** The expression of the ferroptosis-associated genes *GPX4*, *SLC7A11*, and *SLC3A2* was downregulated under JQ1 (10 μM, 24 h) treatment. The gene expression values were normalized to GAPDH expression. An equal volume of DMSO was used as the control. The expression value of the DMSO group was normalized to 1. *P < 0.05, **P < 0.01, ***P < 0.001. The data represent the results of at least three independent experiments

Next, we explored whether JQ1 induces ferroptosis via mechanisms other than ferritinophagy. We performed Kyoto Encyclopedia of Genes and Genomes (KEGG) pathway and Gene Ontology (GO) analyses to predict the potential functions of BRD4. However, none of the pathways recognized in KEGG were directly related to ferroptosis (Fig. 8a). GO analysis identified regulation of transcription as one term related to possible functions of BRD4 (Fig. 8b). Because BRD4 has been reported as a super-enhancer of gene transcription^25^, we investigated whether BRD4 regulates the transcription of ferroptosis-associated genes *GPX4* and the expression of solute carrier family 7 member 11 (*SLC7A11*) and solute carrier family 3 member 2 (*SLC3A2*), which inhibit ferroptosis via the production of reduced glutathione^8^. The results showed that, when BRD4 expression was knocked down, the expression of *GPX4*, *SLC7A11*, and *SLC3A2* was downregulated (Fig. 8c). Furthermore, BRD4 expression was positively related to *GPX4*, *SLC7A11*, and *SLC3A2* expression in pan-cancer patients (Fig. 8d). Then we investigated the expression of these genes under JQ1 treatment. As expected, the expression levels of *GPX4*, *SLC7A11*, and *SLC3A2* were decreased under JQ1 treatment (Fig. 8e). Thus we inferred that JQ1 may induce ferroptosis by controlling the transcription of ferroptosis-associated genes regulated by BRD4. Moreover, we explored whether JQ1 could also regulate autophagy-associated genes via this mechanism. We found that the protein levels of ATG5 and LAMP1 were increased under treatment with JQ1; however, we did not observe this phenomenon in ribosomal protein S6 kinase (S6K), a downstream effector of mammalian target of rapamycin (Fig. S4f). Furthermore, we found that BRD4 expression was negatively associated with both *ATG5* and *LAMP1* expression in pan-cancer patients (Fig. S4g), indicating that JQ1 increases the expression of ATG5 and LAMP1 via the suppression of BRD4 expression, subsequently inducing ferritinophagy. In summary, JQ1 induces ferroptosis by controlling ferroptosis-associated genes regulated by BRD4 and may activate ferritinophagy by increasing the ATG5 and LAMP1 expression.

### JQ1 regulates ferritinophagy and the expression of ferroptosis-associated genes via epigenetic suppression of BRD4

**Fig. 9:**
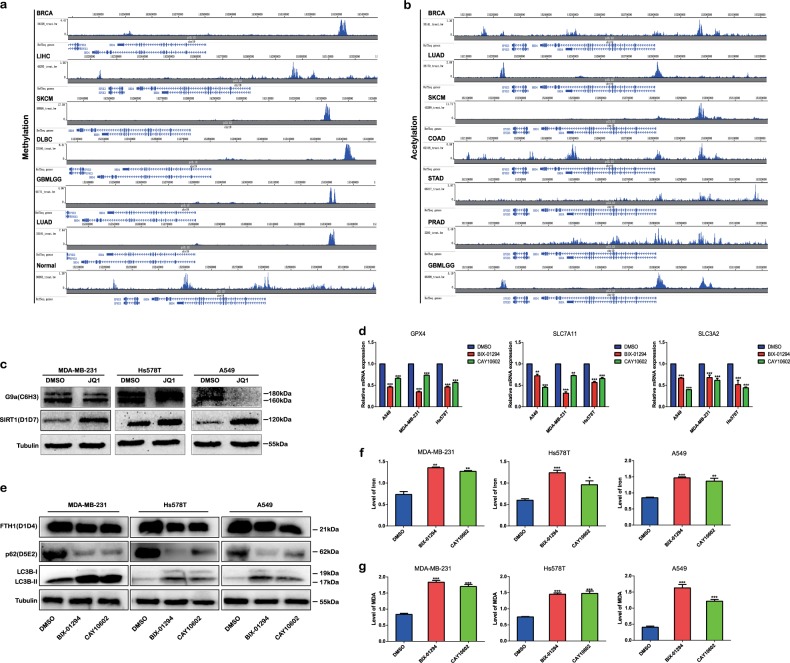
JQ1 regulates ferritinophagy and the expression of ferroptosis-associated genes via epigenetic suppression of BRD4. **a**, **b** Enhancement of H3K4me3 (**a**) and H3K27ac (**b**) at loci upstream of BRD4 in cancer cells from ENCODE (www.encodeproject.org/). **c** Under treatment with JQ1 (10 μM, 24 h), the expression of the histone lysine methyltransferase G9a was suppressed and expression of the histone deacetylase SIRT1 was increased in the MDA-MB-231, Hs578T, and A549 cancer cell lines. **d** The expression of the ferroptosis-associated genes *GPX4*, *SLC7A11*, and *SLC3A2* was downregulated under treatment with G9a inhibitor BIX-01294 (10 μM, 24 h) or SIRT1 activator CAY10602 (5 μM, 24 h). An equal volume of dimethyl sulfoxide (DMSO) was used as the control. The expression values were normalized to GAPDH expression. The expression value of the DMSO group was normalized to 1. **e** Expression of the autophagy markers LC3B-II/LC3B-I was increased and that of SQSTM1 and FTH1 was decreased under treatment with BIX-01294 (10 μM, 24 h) or CAY10602 (5 μM, 24 h). **f**–**g** The levels of iron (**f**) and reactive oxygen species (ROS) (**g**) were increased under treatment with BIX-01294 (10 μM) or CAY10602 (5 μM). An equal volume of DMSO was used as the control, and levels of iron and ROS were assessed after the application of the drugs for 24 h. *P < 0.05, **P < 0.01, ***P < 0.001. The data represent the results of at least three independent experiments

Finally, we investigated the mechanism by which JQ1 regulates BRD4 during ferroptosis. Since epigenetic regulatory mechanisms, including histone modification and DNA methylation, have been identified as important gene expression regulatory mechanisms^26,27^, we explored whether BRD4 could be epigenetically regulated. We identified enrichment of the H3K4me3 or H3K27ac peaks at upstream of BRD4 in cancer cells (Fig. 9a, b). Therefore, upregulation of BRD4 expression in cancer may result from histone methylation or acetylation. As the histone lysine methyltransferase G9a and the histone deacetylase SIRT1 have been reported to interact with BRD4 and is regulated by JQ1^28^, we explored whether JQ1 induces ferroptosis via the regulation of BRD4 by controlling the expression of G9a and SIRT1. As expected, the expression of G9a was downregulated and the expression of SIRT1 was upregulated with JQ1 treatment (Fig. 9c). Furthermore, treatment with the G9a inhibitor BIX-01294 or the SIRT1 activator CAY10602 (Fig. 9d) resulted in decreased expression of the ferroptosis-associated genes *GPX4*, *SLC7A11*, and *SLC3A2*. In addition, we found that, under treatment with BIX-01294 or CAY10602, autophagy markers LC3B-II/LC3B-I was increased but the levels of p62 and FTH1 were decreased (Fig. 9e). Finally, the levels of iron (Fig. 9f) and ROS (Fig. 9g) were increased under treatment with BIX-01294 or CAY10602, indicating that the initiation of ferritinophagy or alteration of ferroptosis-associated genes by JQ1 may be mediated via suppression of BRD4 through the inhibition of G9a-induced histone methylation or enhancement of SIRT1-induced histone deacetylation.

In addition, we explored whether BRD4 is epigenetically regulated via DNA methylation. The specific data, including the numbers of patients and healthy subjects with the corresponding *P* values, are listed in Table 2. BRD4 methylation levels at the cg17726535 locus were lower in cancer tissues than in normal tissues in BLCA, BRCA, COAD, KIRP, and prostate adenocarcinoma (Fig. S2a). In addition, BRD4 methylation levels at the cg19287817 locus were found to be lower in cancer tissues than in normal tissues in BLCA, BRCA, cholangiocarcinoma, KIRC, KIRP, liver hepatocellular carcinoma, LUAD, LUSC, pheochromocytoma and paraganglioma, and thyroid carcinoma (Fig. S2c). Moreover, in pan-cancer patients, the levels of DNA methylation at both the cg17726535 (Fig. S2b) and the cg19287817 loci (Fig. S2d) were lower in cancer tissues than in normal tissues. Thus JQ1 may also induce ferroptosis by inhibiting the BRD4 expression through the enhancement of DNA methylation.

**Table 2:** Number of samples and corresponding significance of DNA methylation of BRD4

Methylated site	Type of cancer	Number of patients	Number of healthy subjects	Significance
cg17726535	BLCA	407	21	***
	BRCA	726	93	****
	COAD	396	45	*
	KIRP	274	45	**
	PRAD	499	50	*
	Pan-Cancer	8612	742	**
cg19287817	BLCA	413	21	****
	BRCA	790	98	****
	CHOL	36	9	****
	KIRC	319	160	****
	KIRP	275	45	*
	LIHC	377	50	****
	LUAD	458	32	***
	LUSC	372	43	****
	PCPG	181	3	****
	THCA	514	56	****
	Pan-Cancer	8767	750	****

*p < 0.05; **p < 0.01; ***p < 0.001; ****p < 0.0001; ns no significance

## Discussion

The discovery of new modalities of cancer therapy has become central to medicine^29^. BRD4 has been recognized as an important regulator in tumor biology due to its role in the regulation of gene expression^30^. Indeed, the targeted BRD4 inhibitor JQ1 has been used in cancer therapy and has exhibited promising therapeutic results in cancer^31–34^. However, in some circumstances, cancer cells escape apoptosis induced by JQ1 and cause treatment failure. Therefore, other therapies that can enhance the anticancer effect of JQ1 are urgently needed.

Ferroptosis is a type of programmed cell death that is characterized by the intracellular accumulation of excessive ROS and iron^35^. In a recent study, nanotargeting of withaferin effectively induced ferroptosis and exhibited satisfactory effects in neuroblastoma treatment^20^. In another study, anticancer drugs such as lapatinib and siramesine were reported to induce ferroptosis^36^, indicating that ferroptosis participates in the anticancer effect of drugs. However, whether JQ1 induces ferroptosis in cancer cells is unknown.

**Fig. 10:**
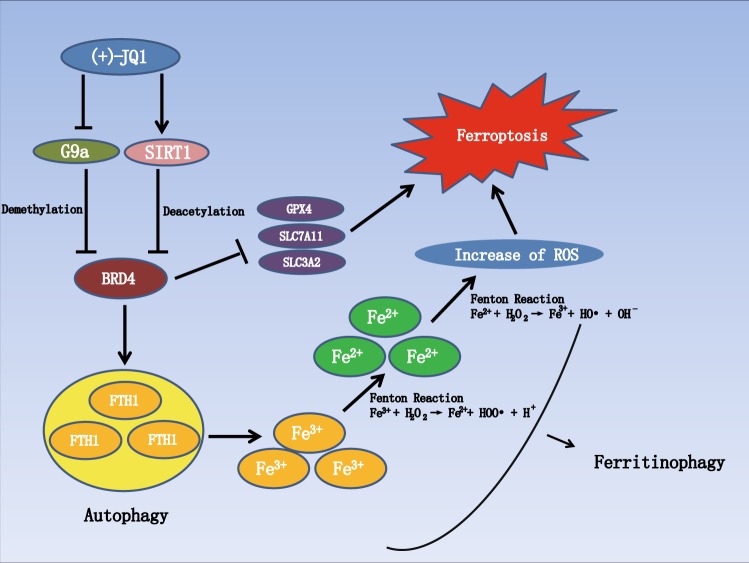
**Schematic diagram of ferroptosis induced by JQ1**

In this study, we analyzed the correlation between JQ1 and ferroptosis. We found that apoptosis is not the only type of death induced by JQ1. Further experiments showed that ferroptosis is involved in JQ1-induced cell death. Treatment with JQ1 and RSL3, erastin, or sorafenib produced a satisfactory anticancer effect, suggesting that the combination of JQ1 with ferroptosis inducers could become a new therapeutic modality. However, ferroptosis occurred not only in cancer cells but also in normal cells. This finding is not novel, and in previous studies, ferroptosis was found to occur in nerve cells and kidney epithelial cells^37,38^, indicating that the therapeutic window of ferroptosis inducers in cancer should be carefully considered. Furthermore, we found that JQ1 induced ferroptosis via the activation of ferritinophagy. This is interesting because the regulation of ferritinophagy by BRD4 has not been reported before. However, since autophagy plays a double-edged role in cancer cells^39^, the possibility that JQ1-induced autophagy could enhance the growth of cancer cells should be considered. In this study, autophagy acted as both a driver in cancer cells and an inducer of ferroptosis under JQ1 treatment. Moreover, consistent with the fact that BRD4 could recruit other transcription factors to the acetylated histone to enhance gene expression^40^, we found that the expression levels of the ferroptosis-associated genes *GPX4*, *SLC7A11*, and *SLC3A2* were decreased under treatment with JQ1. However, the specific mechanisms by which BRD4 regulates the expression of *GPX4*, *SLC7A11*, and *SLC3A2* need further exploration. Finally, we found that JQ1 treatment induced ferroptosis by suppressing BRD4 expression via the inhibition of the histone methylase G9a or activation of the histone deacetylase SIRT1 as there are enhanced H3K4me3 and H3K27ac levels at upstream of BRD4 in cancer. This is consistent with the results of a previous study in which the G9a inhibitor BIX-01294 induced autophagy-associated cell death via intracellular ROS production^41^. In summary, we identified ferroptosis as a new potential approach to eliminate residual cancer cells that are insensitive to apoptosis and then enhance JQ1 therapy (Fig. 10).

Nevertheless, problems remain to be solved. First, ferroptosis occurs in both cancer cells and normal cells^12,42^. Therefore, safety and side effects should be considered during treatment with ferroptosis inducers. Second, whether JQ1 regulates ferroptosis via other mechanisms requires further study. This report offers new insights into both the JQ1 and ferroptosis and illuminates new approaches for cancer therapy.

## Material and methods

### Public data access and analysis

Genome-wide BRD4 expression profiles for pan-cancer and patient survival analyses were downloaded from TCGA (https://tcga-data.nci.nih.gov/). BRD4 expression profiles in cell lines were downloaded from the Broad Institute CCLE (https://portals.broadinstitute.org/ccle/about). BRD4 expression was dichotomized using the median expression level as the cutoff to define “high expression” as an expression level at or above the median versus “low expression” as an expression level below the median. Unpaired Student’s *t* tests were applied to determine significant differences between tumor and normal samples. Analysis of variance was applied to examine differences between groups. GO term enrichment and KEGG pathway data were analyzed using the DAVID Functional Annotation Bioinformatics Microarray Analysis tool (https://david.ncifcrf.gov/). Pairwise Pearson correlations between the expression of BRD4 and that of all genes were examined. Data on BRD4 regulation by H3K27ac and H3K4me3 were obtained from ENCODE (www.encodeproject.org/). DNA methylation profiles were determined experimentally using the Illumina Infinium HumanMethylation450 Beadchip in the TCGA Portal. Beta values were derived at Johns Hopkins University and the University of Southern California TCGA genome characterization center. DNA methylation values, described as beta values, were recorded for each array probe in each sample using the BeadStudio software. DNA methylation beta values are continuous variables with values between 0 and 1 that represent the ratio of the intensity of the methylated bead type to the combined locus intensity. Thus higher beta values represent higher levels of DNA methylation, and lower beta values represent lower levels of DNA methylation.

### Cell culture

The BRCA cell lines MDA-MB-231 and Hs578T were cultured in Dulbecco’s modified Eagle’s medium, and the LUAD cell lines H1299 and A549 were cultured in RPMI-1640 (Gibco, Carlsbad, CA, USA). All media were supplemented with 10% fetal bovine serum (FBS). The nontumorigenic breast epithelial cell line MCF-10A was purchased from Shanghai Zhongqiaoxinzhou Biotech (Shanghai, China (CAS: ZQ0451)) and cultured in corresponding MCF-10A complete medium purchased from Shanghai Zhongqiaoxinzhou Biotech (Shanghai, China (CAS: ZQ1311)). The lung fibroblast cell line IMR90 was purchased from Shanghai Zhongqiaoxinzhou Biotech (Shanghai, China (CAS: ZQ0227)) and cultured in Eagle’s minimal essential medium complete medium purchased from Shanghai Zhongqiaoxinzhou Biotech (Shanghai, China (CAS: ZQ300)). All cells were incubated at 37 °C in humidified air containing 5% CO_2_.

### Cell proliferation assays

Cell proliferation assays were performed using a Cell Counting Kit-8 in accordance with the manufacturer’s instructions (Beyotime, Shanghai, China). Briefly, 5 × 10^3^ cells were seeded into a 96-well plate. Cell proliferation was assessed at 24 h. After the addition of 10 μL of WST-1 reagent per well, the cultures were incubated for 1 h, and absorbance was measured at 490 nm using a microplate reader (BioTek, VT, USA).

### Migration and invasion assays

For migration assays, 5 × 10^4^ cells were seeded into the upper chambers of Transwell culture plates with 8-μm pore membrane inserts (Corning, Shanghai, China). Medium supplemented with 15% FBS was placed in the lower chambers. For the invasion assay, the Transwell chambers were first coated with Matrigel (Catalog Number 356234, Corning) solution. Then cells were seeded into the upper chambers of the plates, and medium supplemented with 15% FBS (600 μL) was added to the lower chambers. After incubation for 24 or 48 h in the migration and invasion assays, respectively, cells that penetrated to the lower surface of the membrane were fixed with 4% paraformaldehyde for 60 min, stained with crystal violet (Sigma, St. Louis, MO, USA) for 60 min, and counted in six randomly chosen fields.

### Transient transfection of cells

For the downregulation of BRD4, ATG5, and ATG7 expression, small interfering RNAs (siRNAs) targeting BRD4, ATG5, and ATG7 were purchased from GenePharma (GenePharma, USA). After culture in 6-well plates for 24 h, cells were transfected with 100 pM siRNA or the corresponding controls using Lipofectamine 2000 (11668-019, Invitrogen, USA) according to the manufacturer’s protocol. Total protein was extracted 72 h after transfection. For GPX4 upregulation, recombinant GPX4 expression plasmids and control plasmids were purchased from GenePharma (GenePharma, USA). After culture in 6-well plates for 24 h, GPX4 expression plasmids or the corresponding control plasmids were transfected into cells using Lipofectamine 2000 according to the manufacturer’s protocol. Total protein was extracted 72 h after transfection. The siRNA sequences are listed in Supplementary Fig. S5a. The sequence of the recombinant plasmid expressing GPX4 is listed in Supplementary Fig. S5b.

### RNA preparation and quantitative real-time PCR (RT-PCR)

Total RNA was extracted using a Total RNA Kit I (OMEGA, CA, USA: R6834-01) in accordance with the manufacturer’s protocol, and cDNA was synthesized using a PrimeScript RT Reagent Kit with gDNA Eraser (Takara Bio, Otsu, Japan). Expression of mRNA was examined by RT-PCR using FastStart Universal SYBR Green Master Mix (Roche, Mannheim, Germany) with gene-specific primers and an ABI StepOne Plus™ Real-time PCR Detection System (Applied Biosystems, Foster City, CA, USA). The expression values were normalized to GAPDH expression. The sequences of the primers (Generay Biotechnology, Shanghai, China) used are listed in Supplementary Figure S5c.

### Transmission electron microscopy

Conventional electron microscopy was performed as previously described^43^. Briefly, cells were fixed with 2.5% glutaraldehyde and postfixed with 1% osmium tetroxide. Then cells were dehydrated in a graded ethanol series and embedded in Embed 812 resin. After mounting on copper grids, the ultrathin sections were double-stained with uranyl acetate and lead citrate. The samples were examined and imaged with an FEI Tecnai Spirit transmission electron microscope.

### Western blot analysis

Cultured cells were harvested and lysed in RIPA buffer containing 1% protease inhibitor. Western blot assays were performed as previously described^44^. The following antibodies were used: anti-BRD4 (ab75898, 1:1000) and anti-GPX4 (ab125066, 1:1000) were purchased from Abcam (Cambridge, MA, USA); anti-G9a (#3306, 1:1000), anti-SIRT1 (#9475, 1:1000), anti-LAMP1 (#9091, 1:1000), anti-S6 kinase (#2708, 1:1000), anti-LC3B (#3868, 1:1000), anti-SQSTM1/p62 (#8205, 1:1000), anti-ATG5 (#12994, 1:1000), anti-ATG7 (#8558, 1:1000), and anti-FTH1 (#4393, 1:1000) were purchased from Cell Signaling Technology; and anti-β-tubulin, used as the internal control, was purchased from Santa Cruz Biotechnology (TX, USA, CAS: KM9002T, 1:1000). After incubation with primary antibodies, membranes were incubated with goat anti-mouse IgG H&L (horseradish peroxidase (HRP)-conjugated) or goat anti-rabbit IgG H&L (HRP-conjugated) for 1.5 h at room temperature. Immunoreactions were then detected with a FluorChem HD2 imager (Protein Simple, USA).

### Immunohistochemistry

Thirteen tumor samples were collected from mice. Tissue sections were dried at 70 °C for 3 h before deparaffinization and rehydration. Subsequently, sections were washed with phosphate-buffered saline (PBS; 3 × 3 min). The washed sections were treated with 3% H_2_O_2_ in the dark for 5–20 min. After washing in distilled water, sections were washed again with PBS (3 × 5 min). Antigen retrieval was performed in citrate buffer (pH 6.0) at 100 °C for 10 min. Each section was incubated overnight at 4 °C with a rabbit polyclonal primary antibody against BRD4 at a 1:100 dilution (Abcam, Cambridge, MA, USA). After washing with PBS (3 × 5 min), each section was further incubated with an anti-rabbit secondary antibody (1:200; Abcam, Cambridge, MA, USA) at room temperature for 30 min. Each section was immersed in 500 μL of a diaminobenzidine working solution at room temperature for 3–10 min after washing with PBS. Finally, the slides were counterstained with hematoxylin and mounted in Crystal Mount medium. BRD4 expression was analyzed and scored independently by two observers based on the intensity and distribution of positively stained tumor cells, which were demarcated by yellow particles in the cytoplasm. The BRD4 staining index was evaluated as follows for BET proteins: no detectable staining in >70% of tumor cell nuclei was considered negative (−), weak staining in ≥30% of tumor cell nuclei was considered weak (+), and strong staining (invisible nucleoli) in >30% of nuclei was considered strong (++)^45^.

### Iron assay

Intracellular ferrous iron levels were determined using an Iron Colorimetric Assay Kit purchased from ScienCell (Cat: 8448). According to the manufacturer’s instructions, cells or tissues (tissues were frozen in liquid nitrogen and ground) were added to iron assay buffer, homogenized on ice, and centrifuged at 13,000 × *g* for 10 min at 4 °C to obtain the supernatant for the assay. A 50-µL sample was incubated with 50 µL of assay buffer in a 96-well microplate for 30 min at 25 °C. Then the sample was incubated with 200 µL of reagent mix in the dark for 30 min at 25 °C, and the absorbance was measured at 590 nm with a microplate reader.

### Lipid peroxidation assessment

Lipid peroxidation was assessed using an MDA Assay Kit (Nanjing Jiancheng Bioengineering Institute, Nanjing, China) according to the manufacturer’s protocol. Briefly, cells or tissues (tissues were frozen in liquid nitrogen and ground) were added to lysis buffer, homogenized on ice, and centrifuged at 1000 × *g* for 15 min at 4 °C to obtain the supernatant. After the protein concentration was measured, 100 µL of sample was incubated with 100 µL of test solution for 40 min at 100 °C. After cooling to room temperature, samples were centrifuged at 4000 × *g* for 10 min to obtain the supernatant, the absorbance of which was measured at 530 nm on a microplate reader. MDA content was expressed as a ratio to the absorbance value of control cells.

### Animal experiments

Animal experiments were approved by the Medical Experimental Animal Care Commission of Harbin Medical University. Female athymic BALB/c nude mice (4–6-week old) were obtained from Beijing Vital River Laboratory Animal Technology Co., Ltd. (Beijing, China). Approximately 1 × 10^7^ cells (A549) in 200 μL of serum-free medium and Matrigel solution were injected directly into the right axilla of each mouse. Tumor growth was measured with calipers every 3 days, and tumor volumes were calculated using the following formula: 1/2 (length × width^2^). When the volume of the tumors was approximately 200 mm^3^, treatment with DMSO, JQ1 (50 mg/kg), or Fer-1 (1 mg/kg) plus JQ1 (50 mg/kg) was administered via intraperitoneal injection daily for 3 and 5 days as a treatment course. The mice were euthanized 18 days after the beginning of the injections, and the tumors were weighed.

### Reagents and application

The following reagents were purchased from MedChem Express (MonmouthJunction, NJ, USA): 3-MA (CA: HY-19312), BIX-01294 (CA: HY-10587), CAY10602 (CA: HY-104073), DFO (CA: HY-B0988), erastin (CA: HY-15763), fer-1 (CA: HY-100579), JQ1 (CA: HY-13030), nec-1 (CA: HY-15760), RSL3 (CA: HY-100218A), sorafenib (CA: HY-10201), and Z-VAD-FMK (CA: 16658).

### Statistical analyses

Data analyses were performed using GraphPad (GraphPad Prism, La Jolla, CA, USA). OS and DFS were calculated as the time from surgery until the occurrence of death or relapse, respectively. DNA expression was dichotomized using a study-specific median expression level as the cutoff to define “high expression” as an expression level at or above the median versus “low expression” as an expression level below the median. Differences between the groups in the in vitro experiments were analyzed using Student’s *t* test. All experiments were performed in triplicate. SPSS 16.0 software (SPSS, Chicago, IL, USA) was used for statistical analysis. All statistical tests were two sided, with *P* < 0.05 considered significant.

## Supplementary information

Supplementary Figure 1

Supplementary Figure 2

Supplementary Figure 3

Supplementary Figure 4

Supplementary Figure 5

supplementary figure legends
