# Supplementary figures and images for: Ferritinophagy is required for the induction of ferroptosis by the bromodomain protein BRD4 inhibitor (+)-JQ1 in cancer cells
Source: Cell Death Dis. 2019 Apr 15;10(5):331. doi: 10.1038/s41419-019-1564-7 (PMC6465411; doi:10.1038/s41419-019-1564-7)

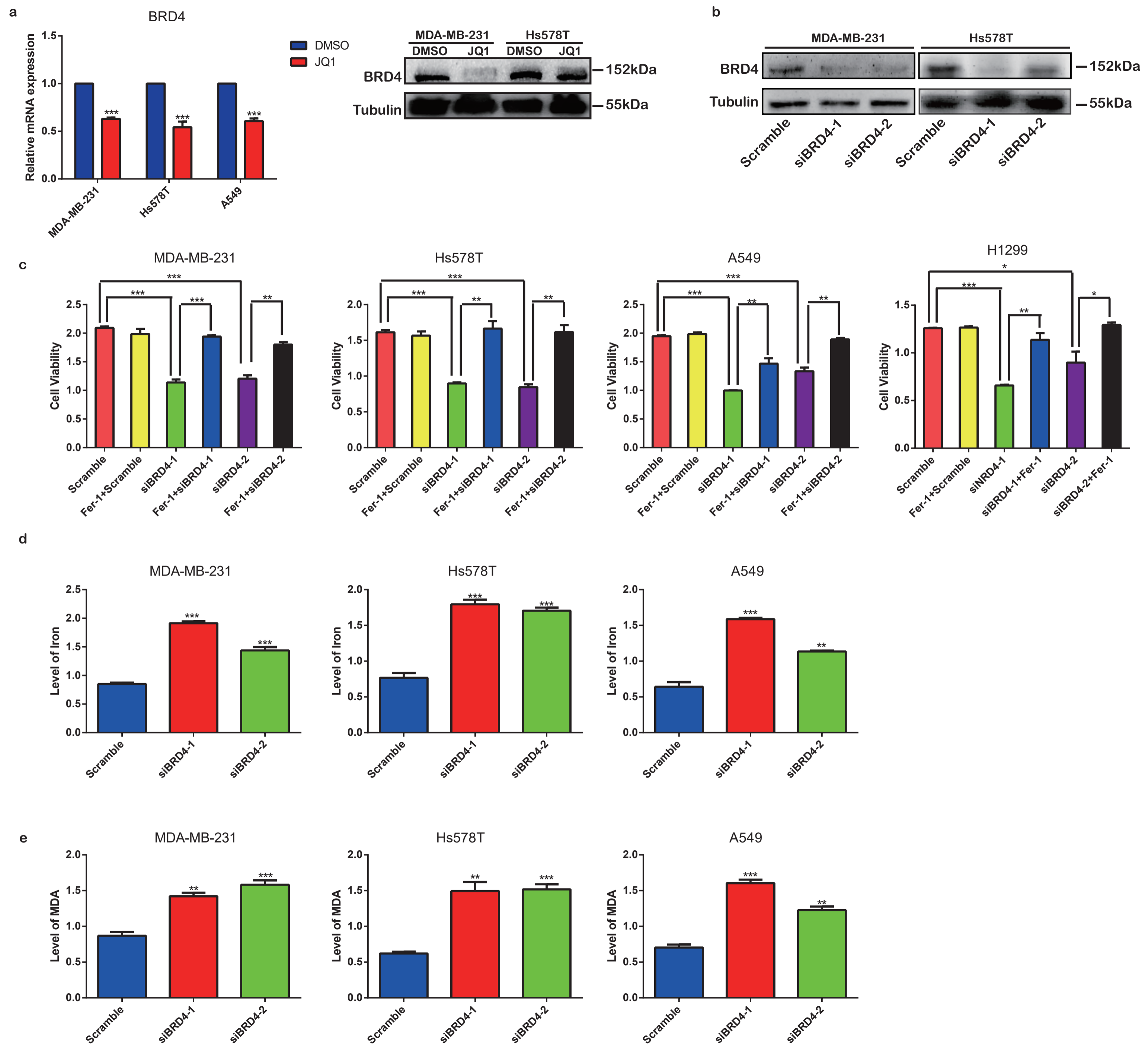

Supplement: Supplementary file 1 — Supplementary Figure 1 [file 41419_2019_1564_MOESM1_ESM.pdf]

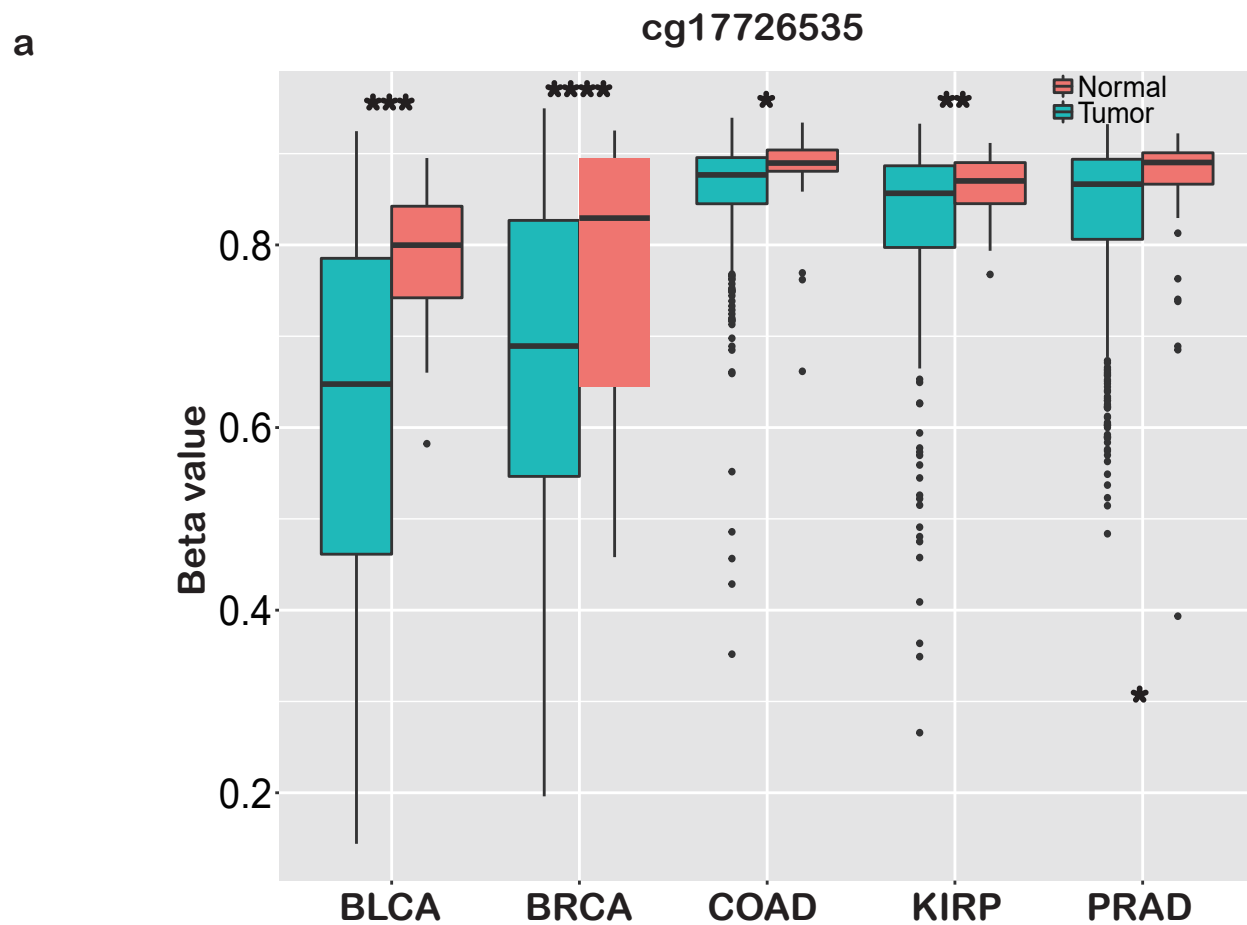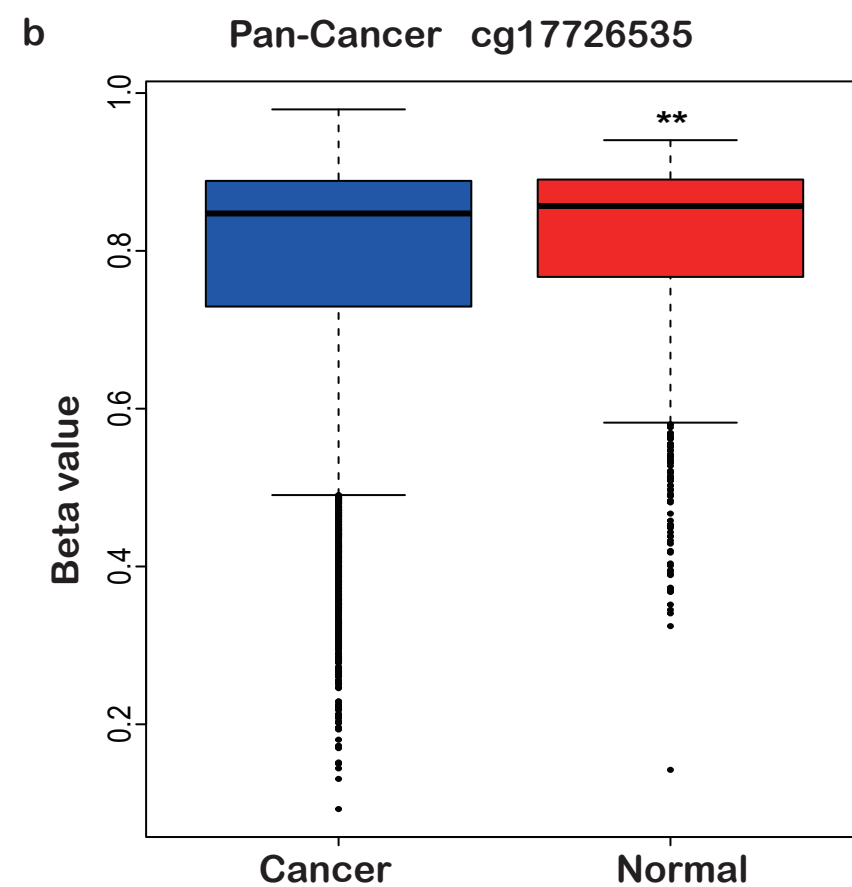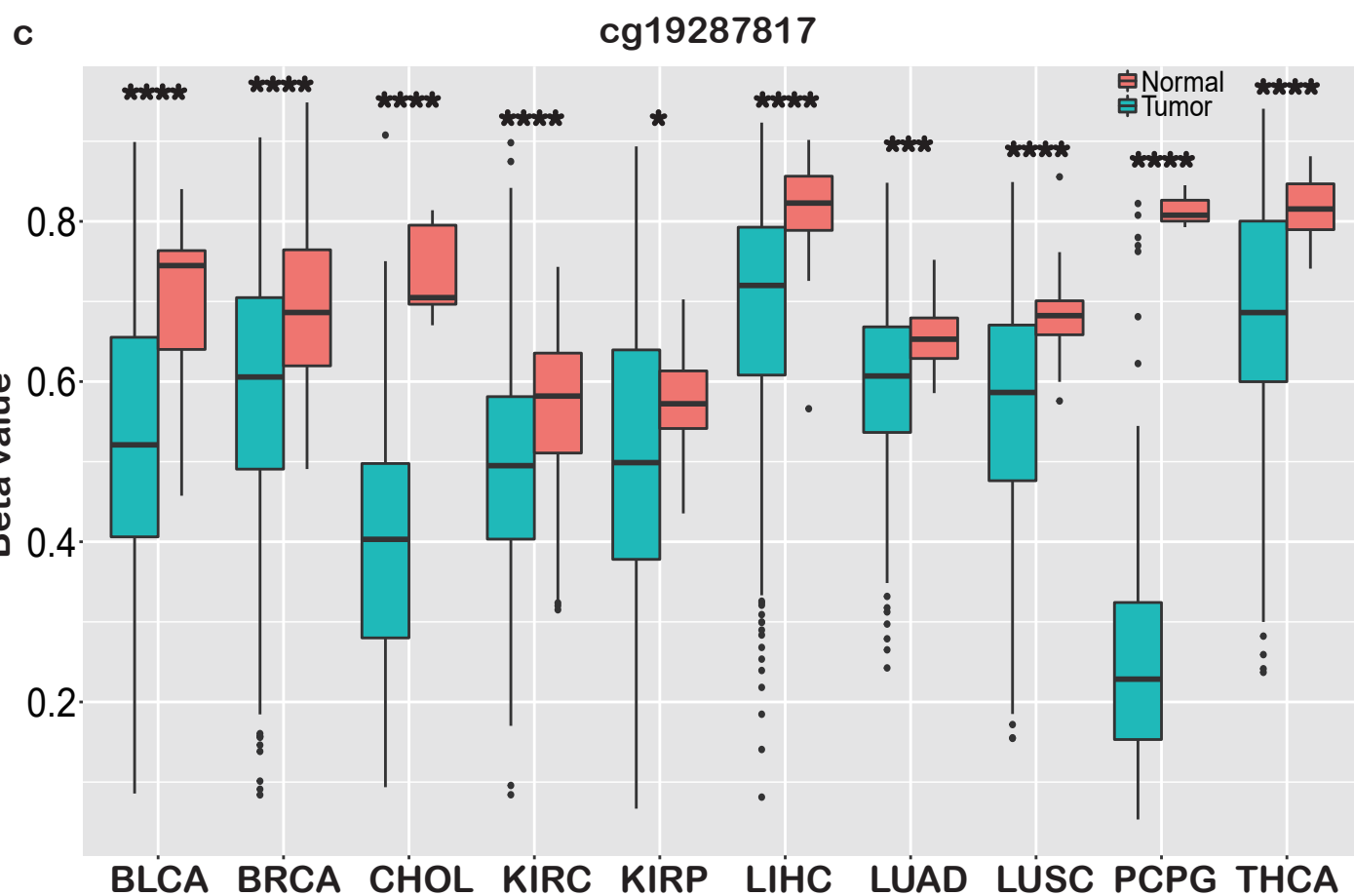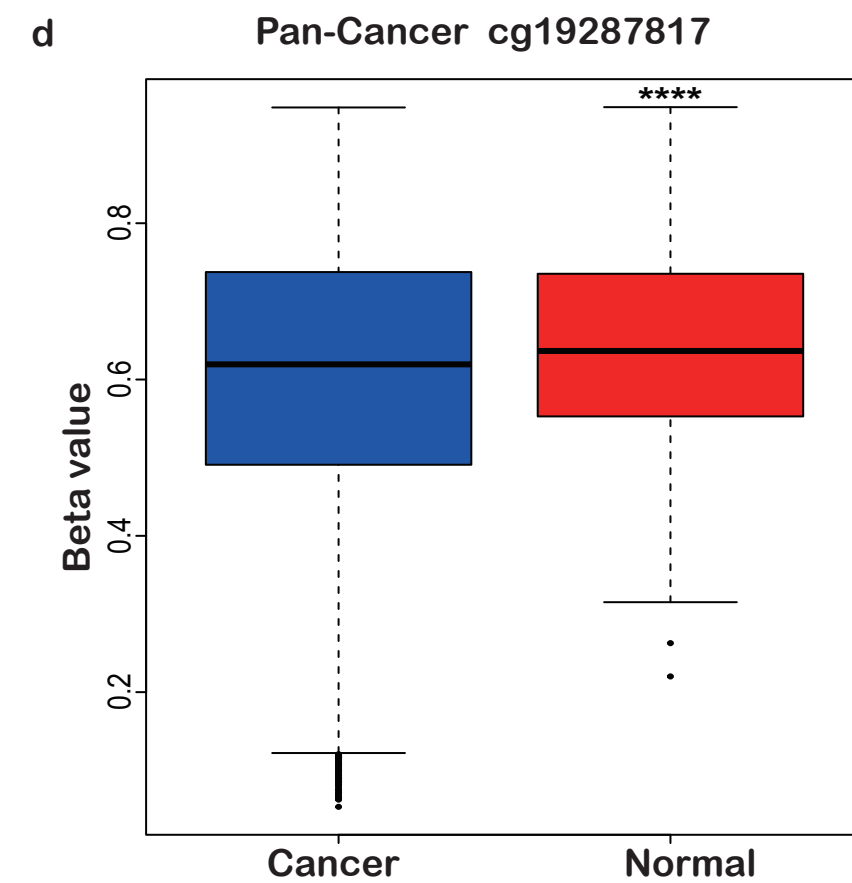

Supplement: Supplementary file 2 — Supplementary Figure 2 [file 41419_2019_1564_MOESM2_ESM.pdf]

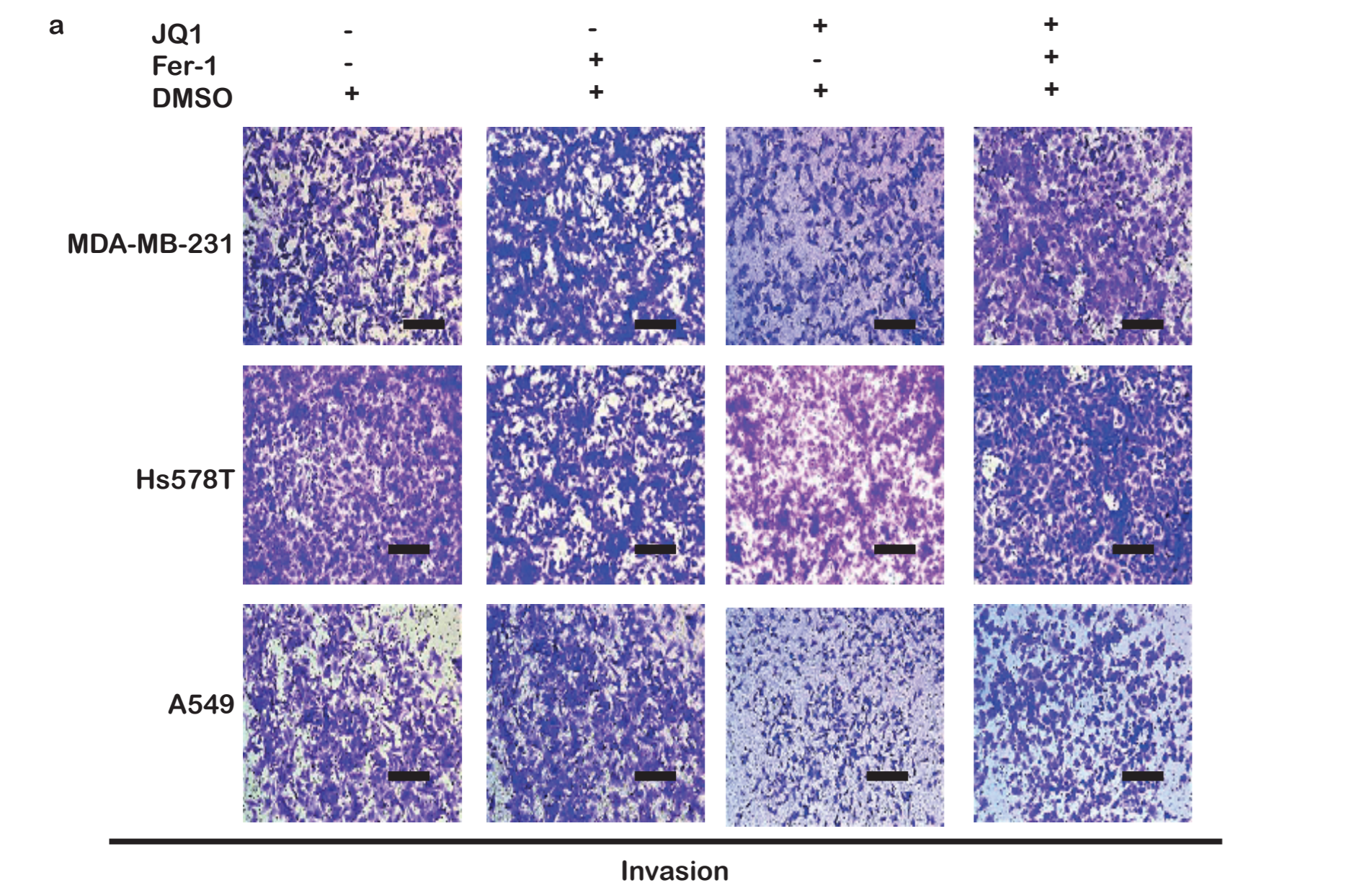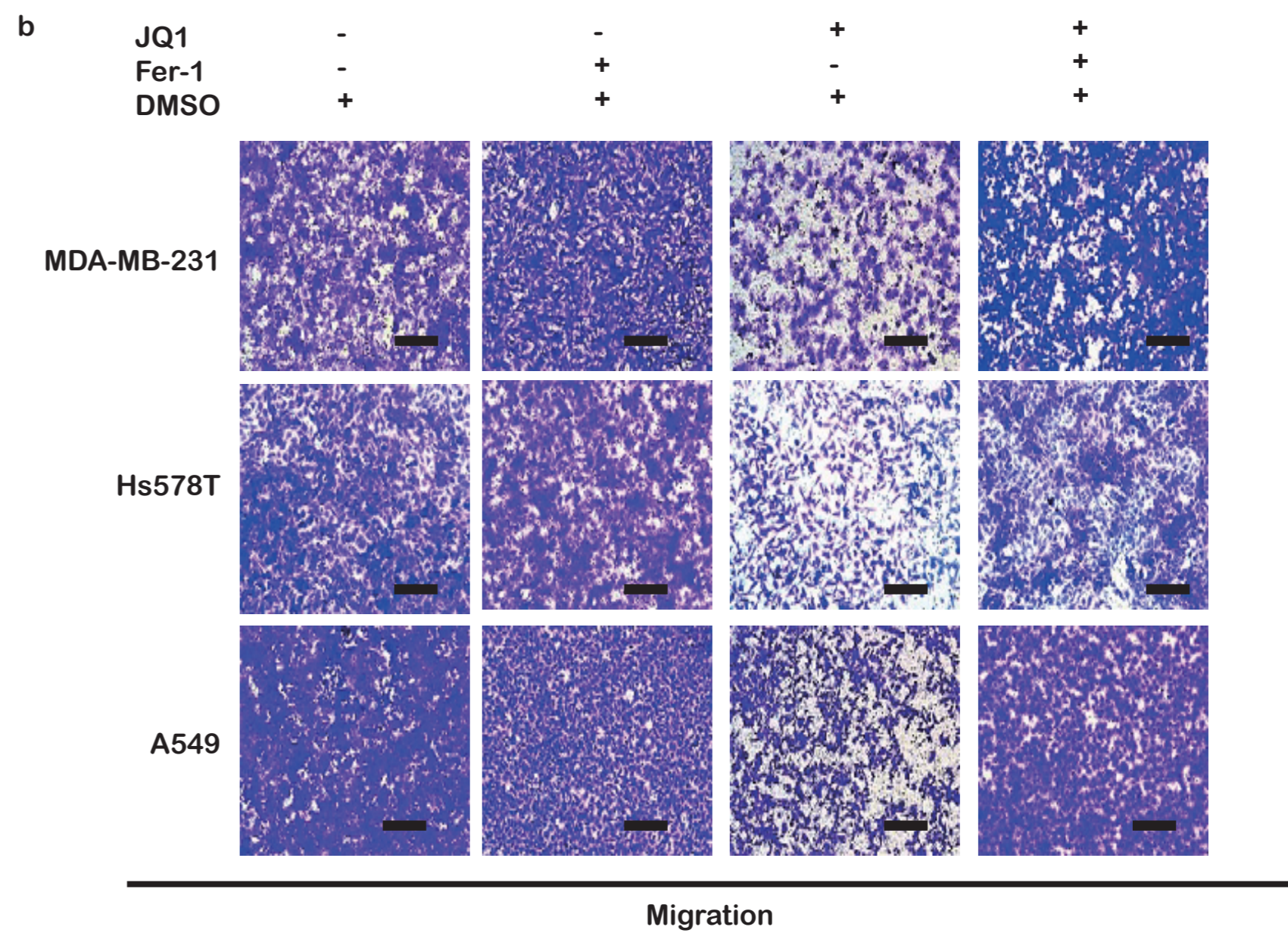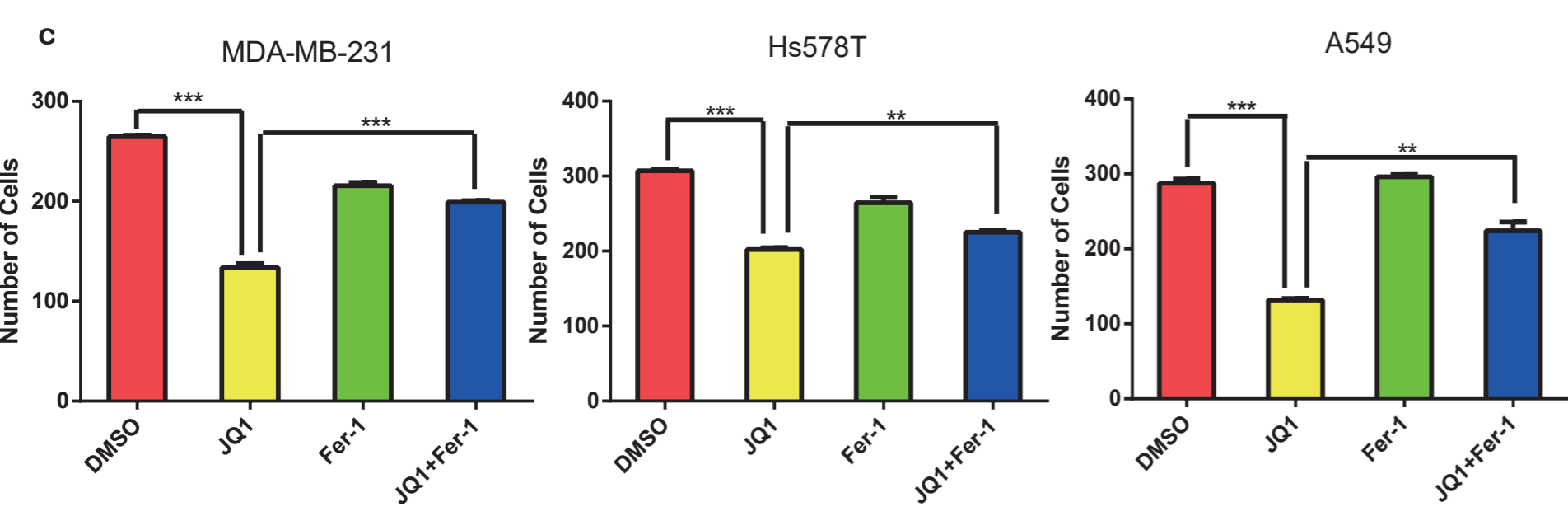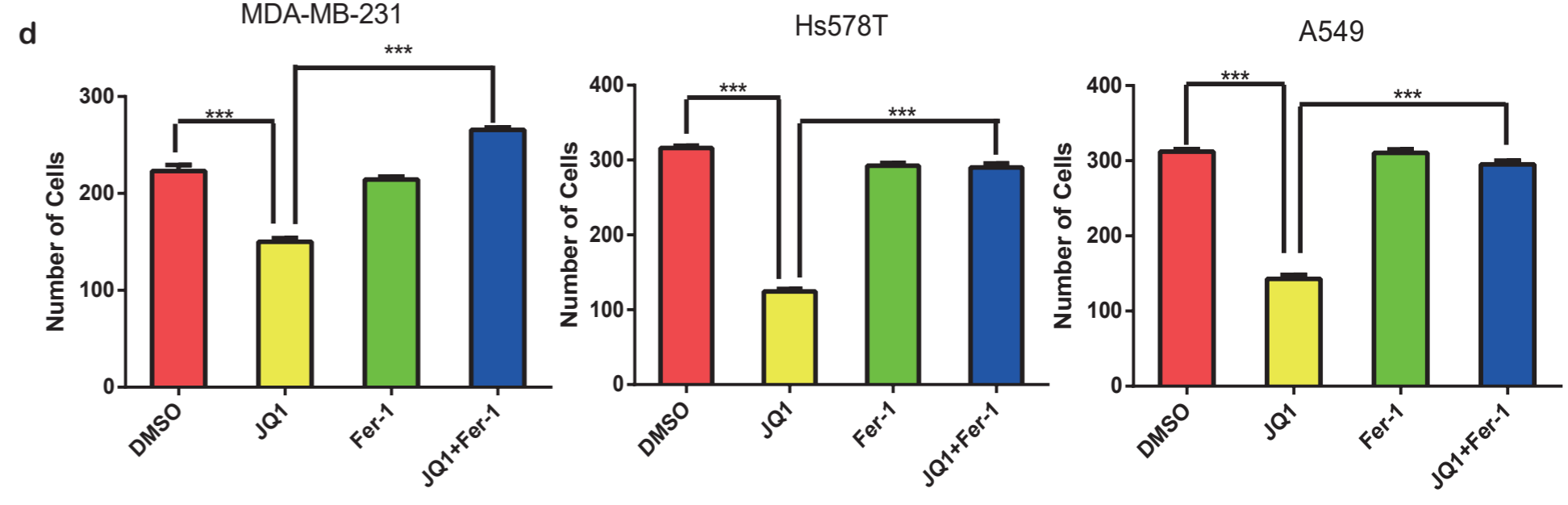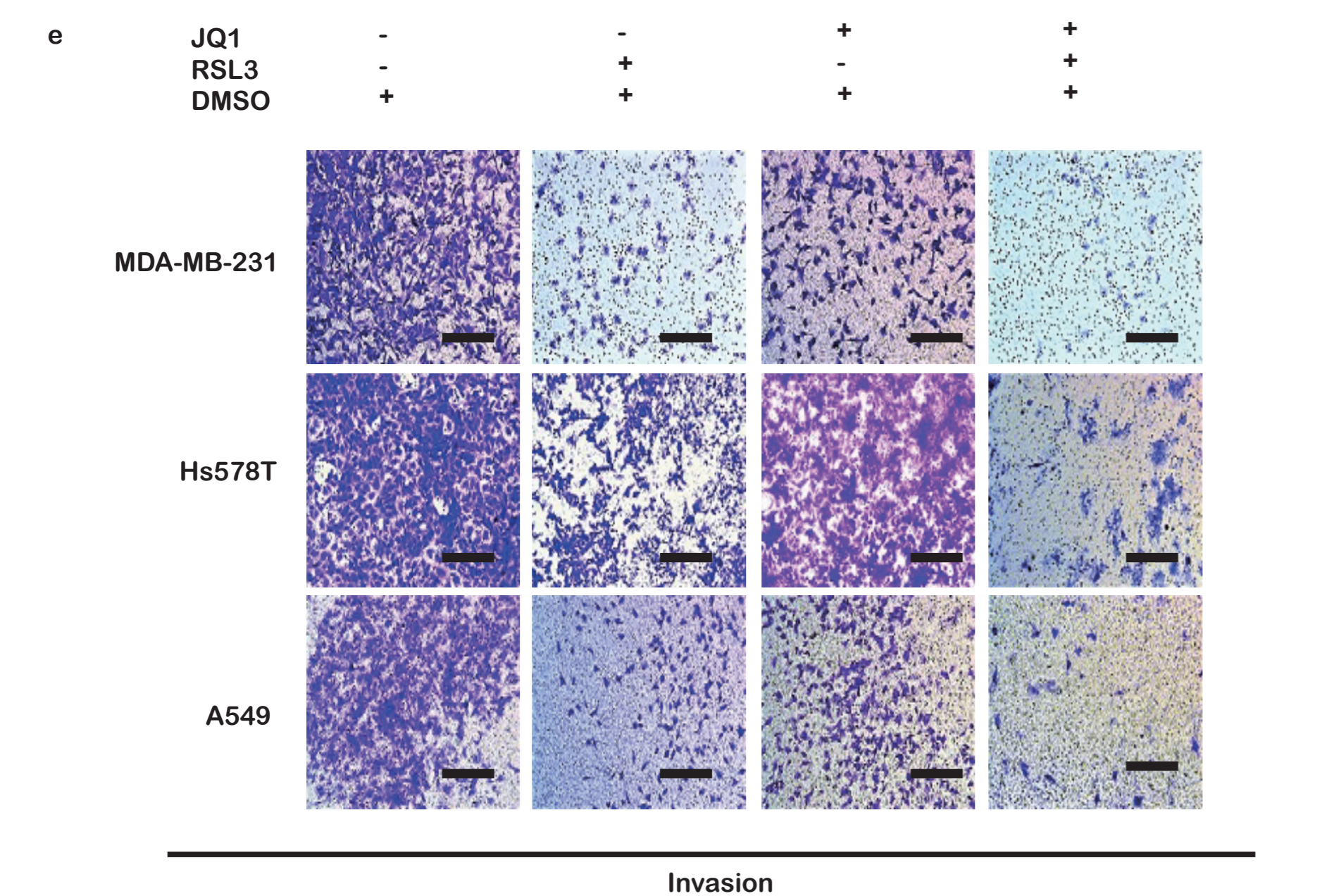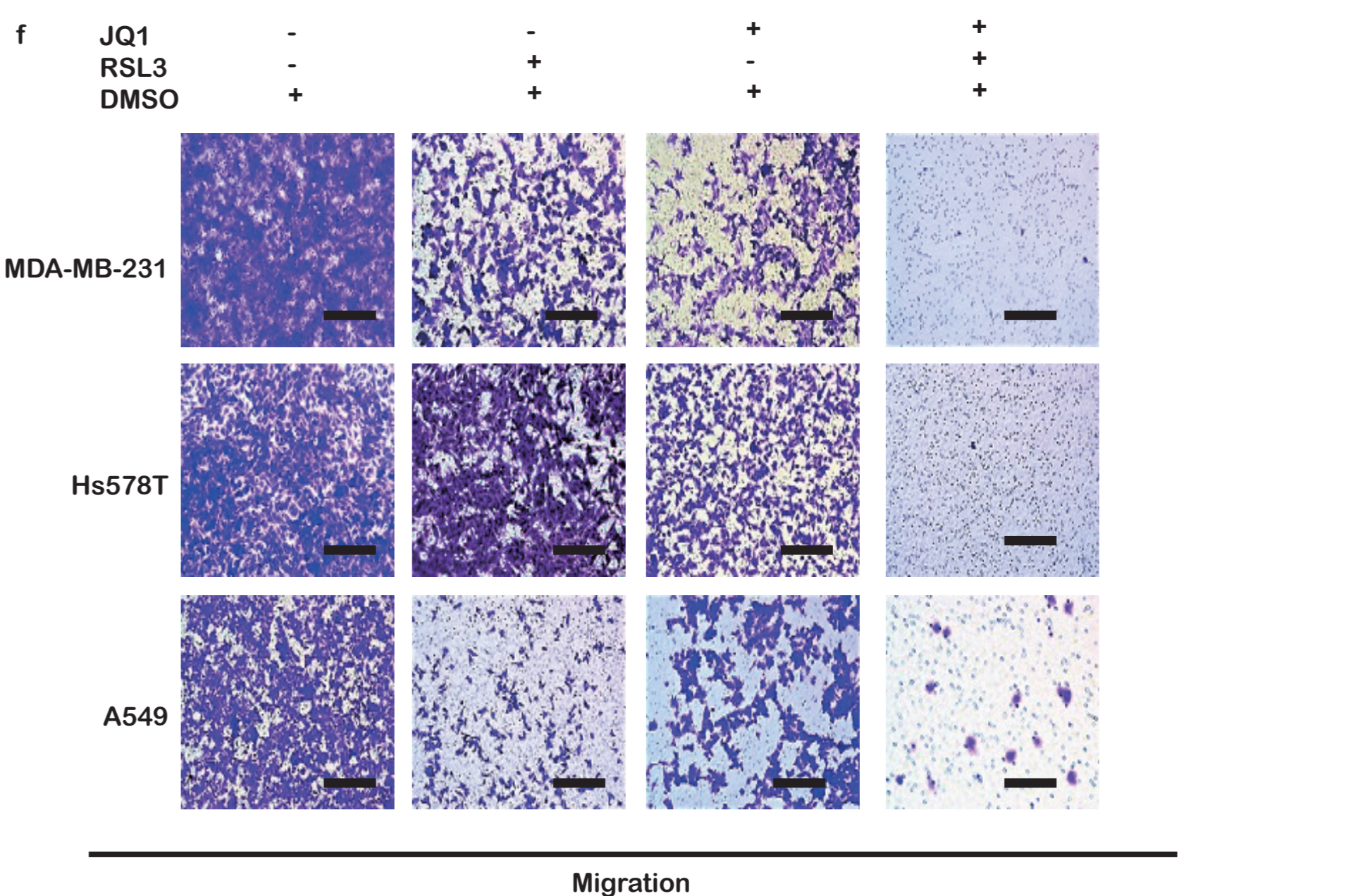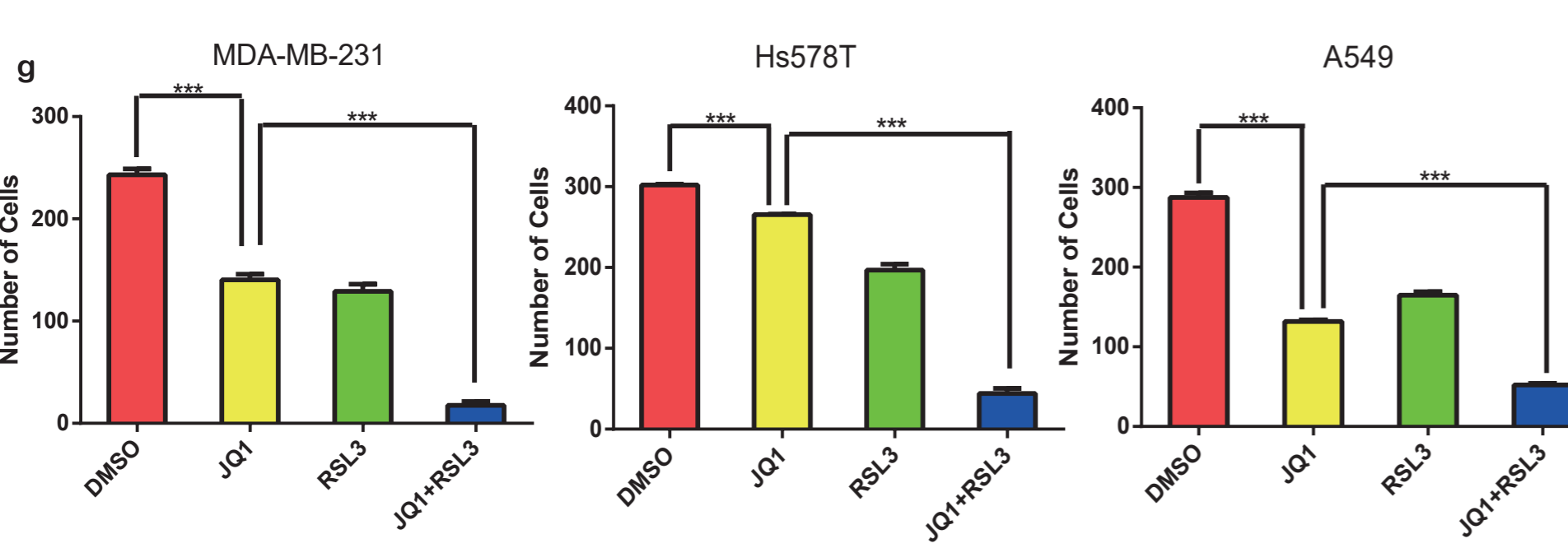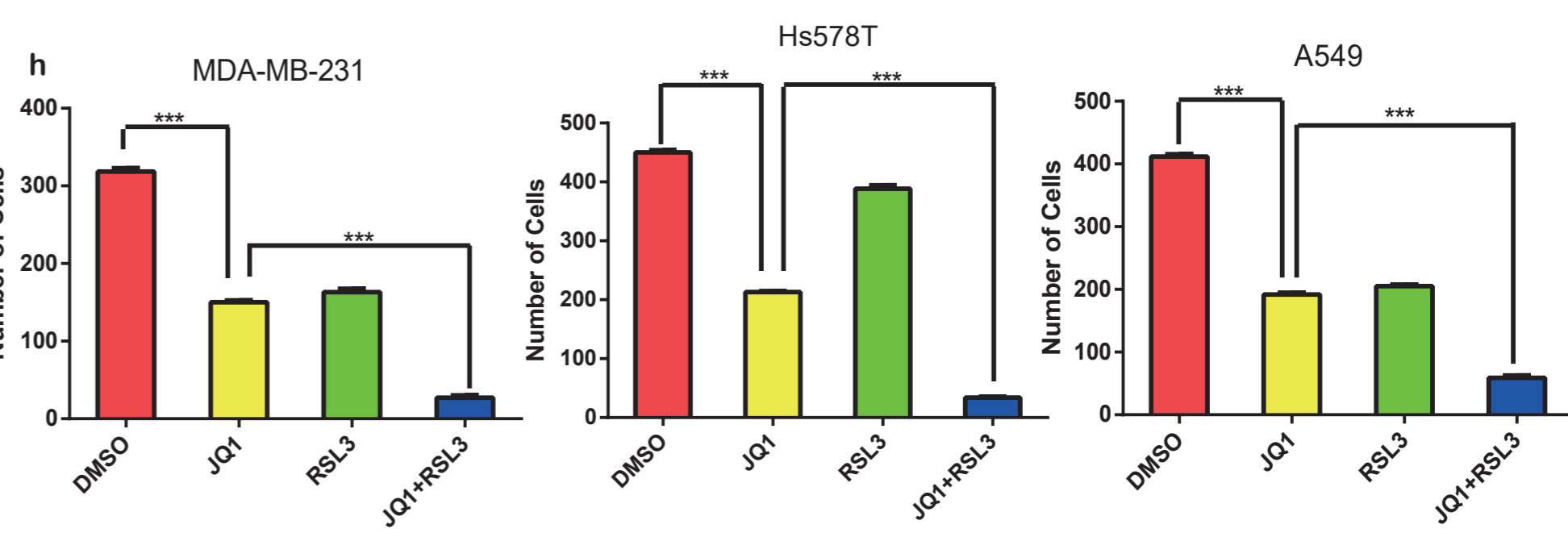

Supplement: Supplementary file 3 — Supplementary Figure 3 [file 41419_2019_1564_MOESM3_ESM.pdf]

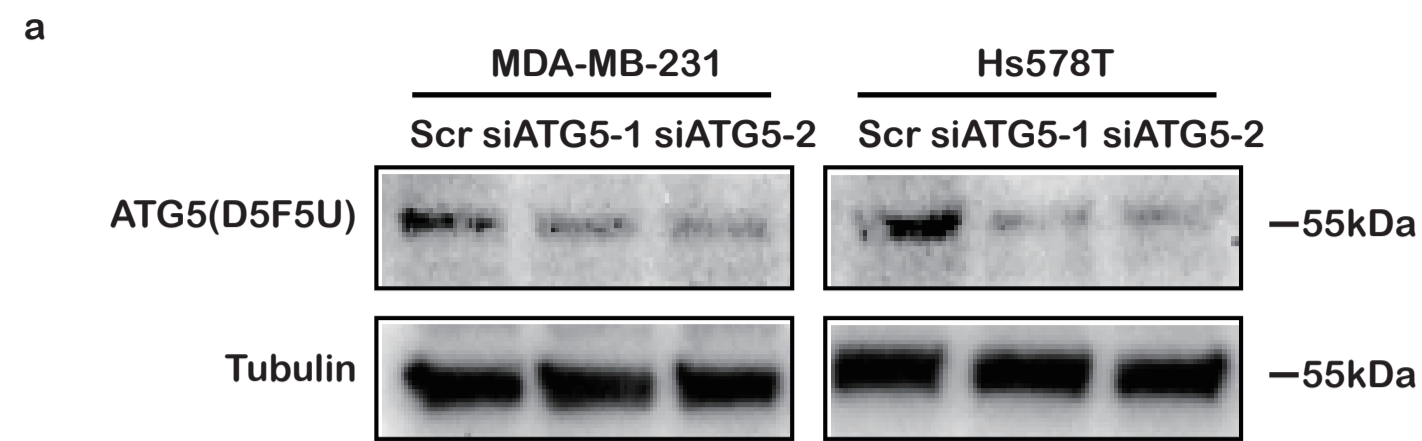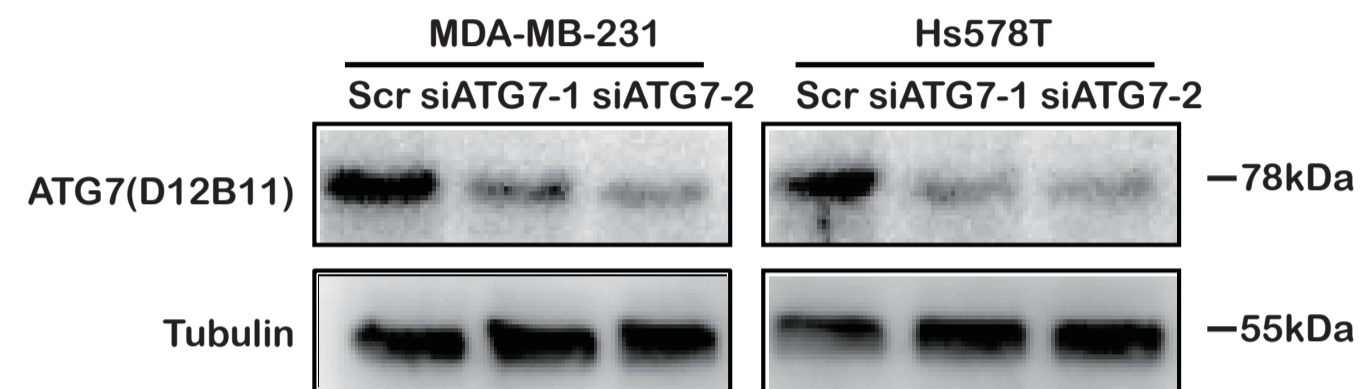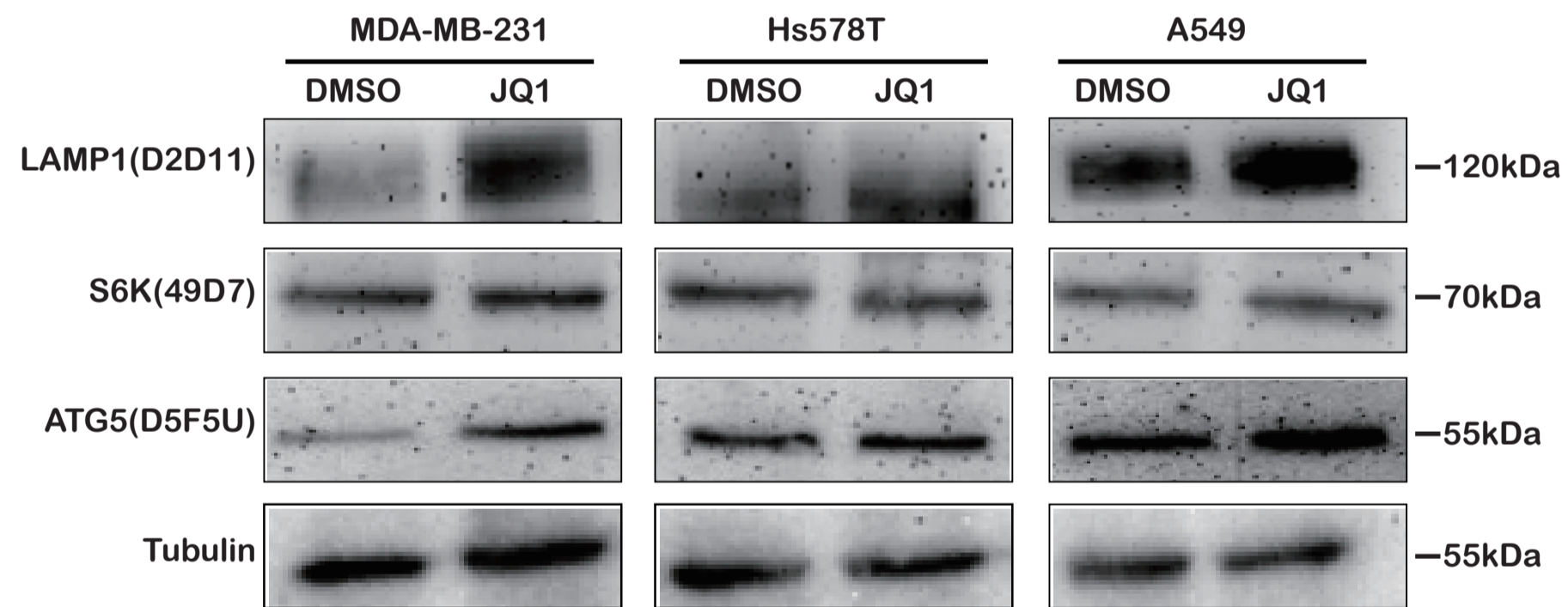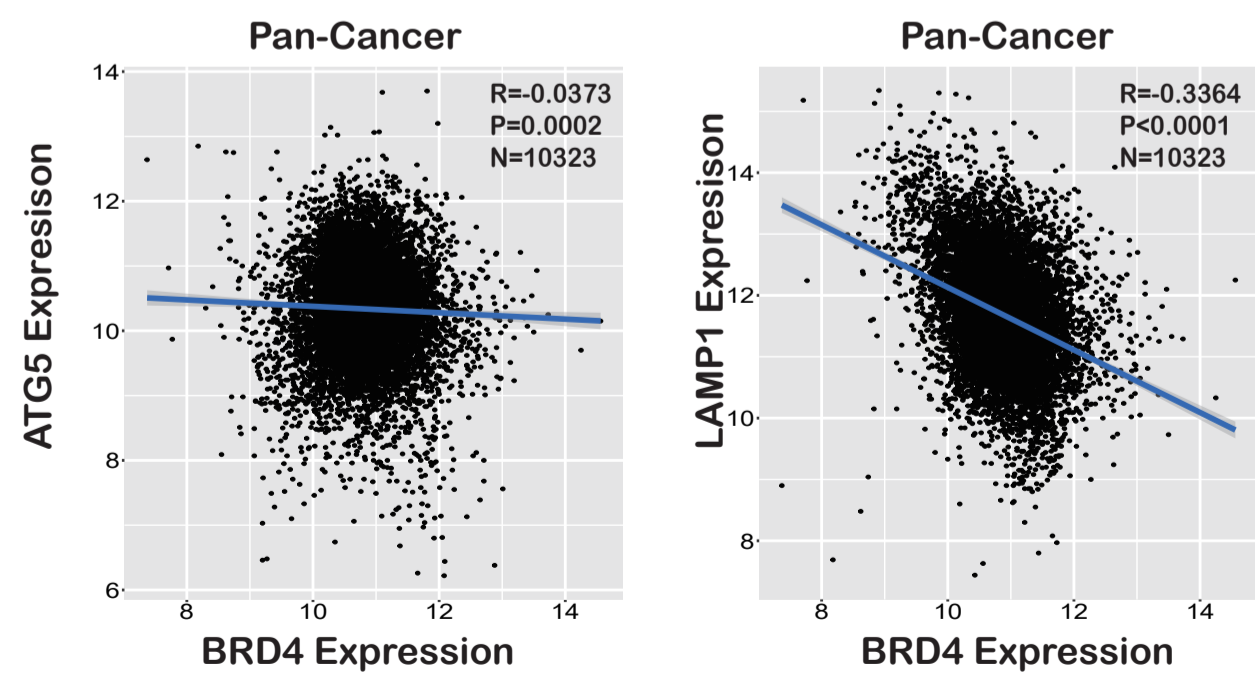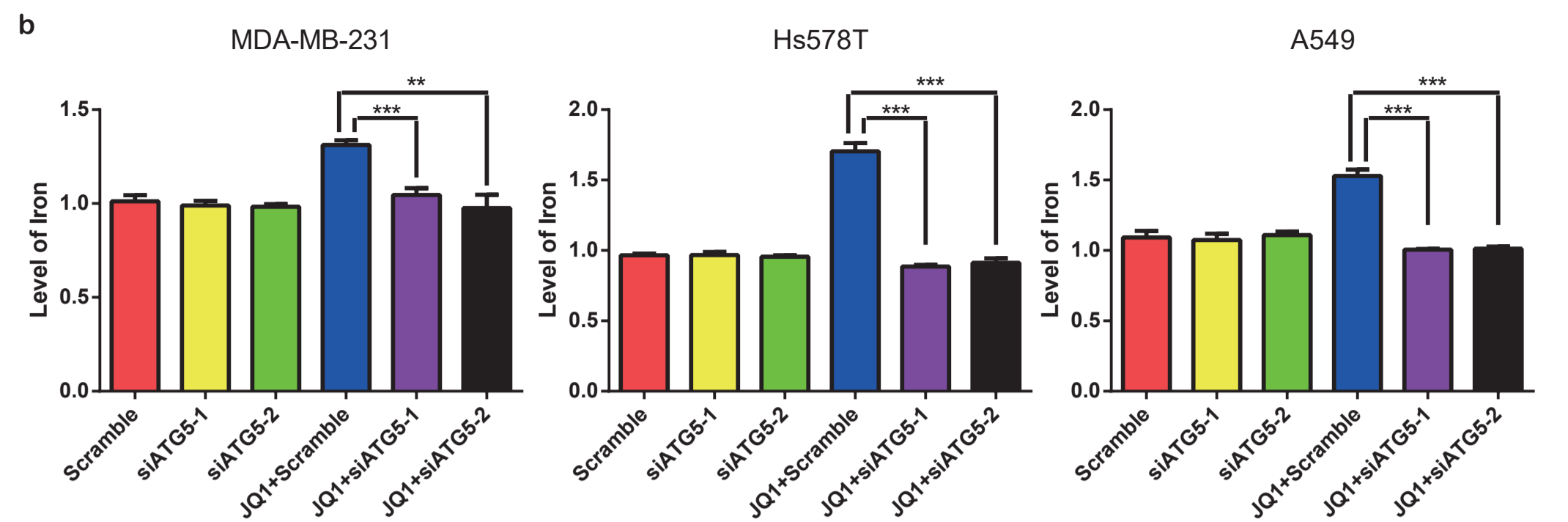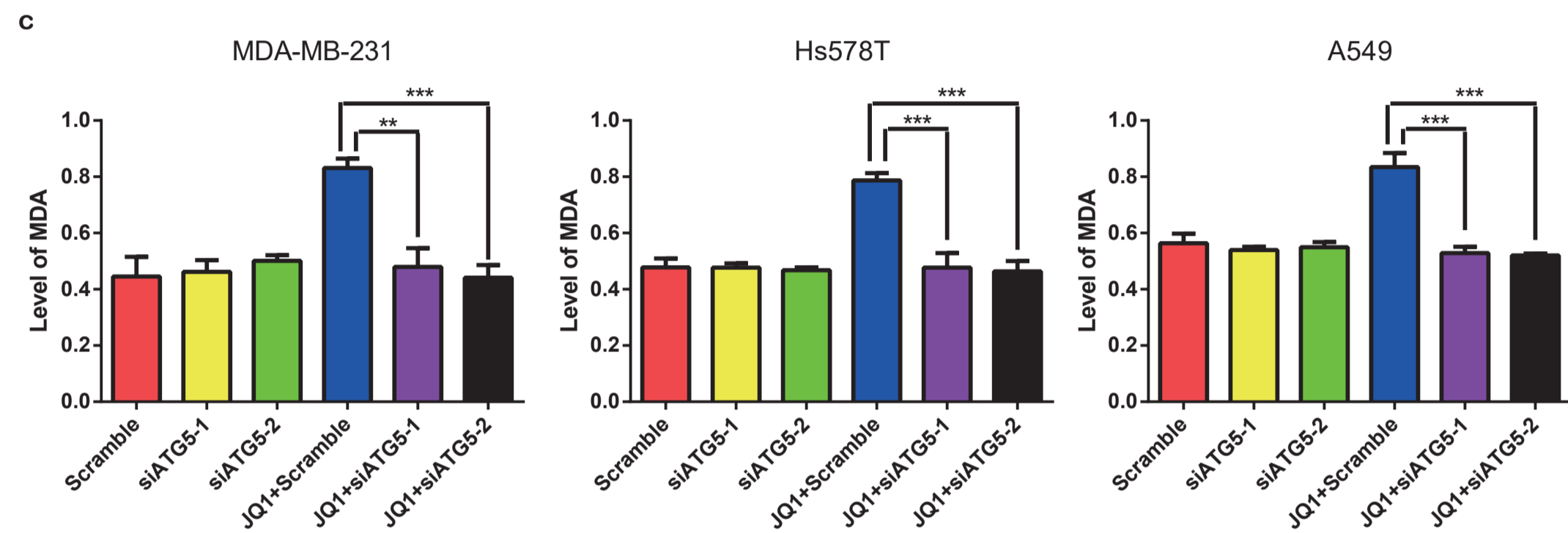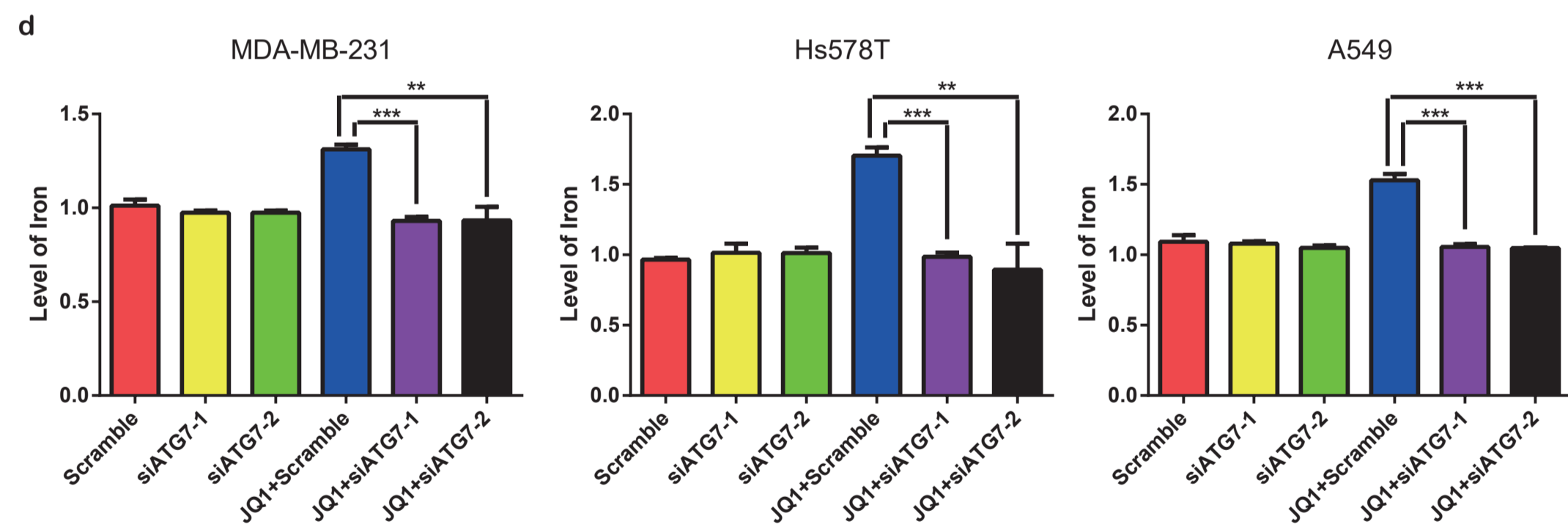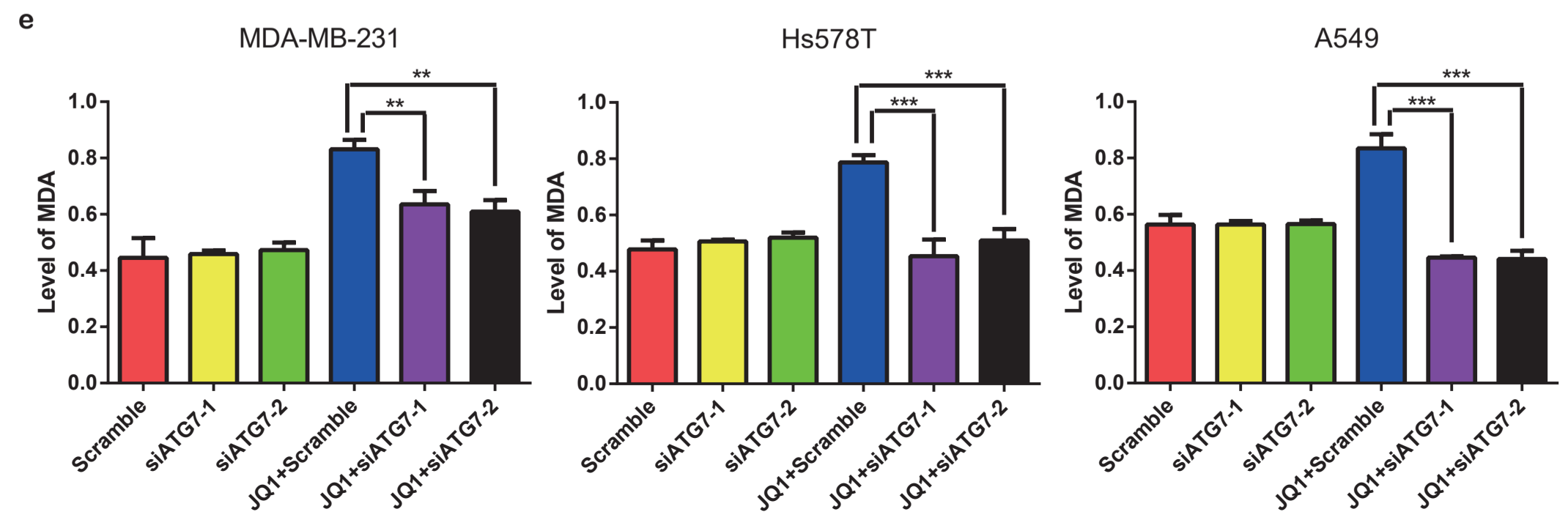

Supplement: Supplementary file 4 — Supplementary Figure 4 [file 41419_2019_1564_MOESM4_ESM.pdf]
