# Supplementary material for: Ferritinophagy is required for the induction of ferroptosis by the bromodomain protein BRD4 inhibitor (+)-JQ1 in cancer cells
Source: Cell Death Dis. 2019 Apr 15;10(5):331. doi: 10.1038/s41419-019-1564-7 (PMC6465411; doi:10.1038/s41419-019-1564-7)
Supplement: Supplementary file 5 — Supplementary Figure 5 [file 41419_2019_1564_MOESM5_ESM.pdf]

a **The sequences of siRNA**

BRD4-siRNAi1 sense: 5'-GCGUUUCCACGGUACCAAATT-3';

BRD4-siRNAi1 anti-sense: 5'-UUUGGUACCGUGGAAACGCTT-3';

BRD4-siRNAi2 sense: 5'-CCGUGAUGCUCAGGAGUUUTT-3';

BRD4-siRNAi2 anti-sense: 5'-AAACUCCUGAGCAUCACGGTT-3';

ATG5-siRNAi1 sense: 5'-GGAAGCAGAACCAUACUAUTT-3';

ATG5-siRNAi1 anti-sense: 5'-AUAGUAUGGUUCUGCUUCCTT-3';

ATG5-siRNAi2 sense: 5'-CCAUCAAUCGGAAACUCAUTT-3';

ATG5-siRNAi2 anti-sense: 5'-AUGAGUUUCCGAUUGAUGGTT-3';

ATG7-siRNAi1 sense: 5'-CCAACAUCCCUGGUUACAATT-3';

ATG7-siRNAi1 anti-sense: 5'-UUGUAACCAGGGAUGUUGGTT-3';

ATG7-siRNAi2 sense: 5'-GCCUCUCUAUGAGUUUGAATT-3';

ATG7-siRNAi2 anti-sense: 5'-UUCAAACUCAUAGAGAGGCTT-3';

Scrambled-siRNA sense: 5'-UUCUCCGAACGUGUCACGUTT-3';

Scrambled-siRNA anti-sense: 5'-ACGUGACACGUUCGGAGAATT-3'.

b **The sequence of recombinant plasmid expressing GPX4**

ATGAGCCTCGGCCGCCTTTGCCGCCTACTGAAGCCGGCGCTGCTCTGTGGGGCTCTGG
CCGCGCCTGGCCTGGCCGGGACCATGTGCGCGTCCCGGGACGACTGGCGCTGTGCGC
GCTCCATGCACGAGTTTTCCGCCAAGGACATCGACGGGCACATGGTTAACCTGGACAA
GTACCGGGGCTTCGTGTGCATCGTCACCAACGTGGCCTCCCAGTGAGGCAAGACCGA
AGTAAACTACACTCAGCTCGTCGACCTGCACGCCCGATACGCTGAGTGTGGTTTGCGG
ATCCTGGCCTTCCCGTGTAACCAGTTCGGGAAGCAGGAGCCAGGGAGTAACGAAGAG
ATCAAAGAGTTCGCCGCGGGCTACAACGTCAAATTCGATATGTTCAGCAAGATCTGCG
TGAACGGGGACGACGCCCACCCGCTGTGGAAGTGGATGAAGATCCAACCCAAGGGCA
AGGGCATCCTGGGAAATGCCATCAAGTGGAACTTCACCAAGTTCCTCATCGACAAGAA
CGGCTGCGTGGTGAAGCGCTACGGACCCATGGAGGAGCCCCTGGTGATAGAGAAGGA
CCTGCCCCACTATTTCTAG

c **The sequences of the primers for Real-time PCR**

BRD4-Forward: 5'-CAAACAGGAGGTGACATAGCG-3';

BRD4-Reverse: 5'-GTCGGAGGAAGAGGACAAGTG-3';

GAPDH-Forward: 5'-CATGTTCGTCATGGGTGTGAA-3';

GAPDH-Reverse: 5'-CGCATGGACTGTGGTCATGAG-3';

GPX4-Forward: 5'-TAGAAATAGTGGGGCAGGTCC-3';

GPX4-Reverse: 5'-CGTCAAATTCGATATGTTCAGC-3';

SLC3A2-Forward: 5'-GAGCCTTGCCTGAGACAAACT-3';

SLC3A2-Reverse: 5'-ACTGGGGAGCATACAAAATCC-3';

SLC7A11-Forward: 5'-GCAACAAAGATCGGAACTGCT-3';

SLC7A11-Reverse: 5'-GCTGGCTGGTTTTACCTCAAC-3'.
