# Supplementary material for: Ferritinophagy is required for the induction of ferroptosis by the bromodomain protein BRD4 inhibitor (+)-JQ1 in cancer cells
Source: Cell Death Dis. 2019 Apr 15;10(5):331. doi: 10.1038/s41419-019-1564-7 (PMC6465411; doi:10.1038/s41419-019-1564-7)
Supplement: Supplementary file 6 — supplementary figure legends [file 41419_2019_1564_MOESM6_ESM.docx]

**Supplementary Figure S1**

**a** Effect of JQ1 (10 μM, 24h) on BRD4 expression. **b** Effect of siBRD4-1 and siBRD4-2 on BRD4 expression. **c** Fer-1 (1 μM) increased the viability of cells with BRD4 knockdown, ratio of knockdown of BRD4 is shown in Fig. S1b. **d** Knockdown of BRD4 increased the levels of iron in MDA-MB-231, Hs578T and A549 cells. **e** Knockdown of BRD4 increased the levels of ROS in MDA-MB-231, Hs578T and A549 cells. An equal volume of DMSO was used as the control, cell viability was assessed after the application of the drugs for 24 h.* *P*<0.05, ** *P*<0.01, *** *P*<0.001. The data represent the results of at least three independent experiments.

**Supplementary Figure S2**

**a-b** Low levels of DNA methylation were identified at the BRD4 cg17726535 locus in patients with various types of cancer (**a**) and in pan-cancer patients (**b**) via the Illumina Infinium HumanMethylation450 platform. **c-d** Low levels of DNA methylation were identified at the BRD4 cg19287817 locus in patients with various types of cancer (**c**) and in pan-cancer patients (**d**) via the Illumina Infinium HumanMethylation450 platform. * *P*<0.05, ** *P*<0.01, *** *P*<0.001, **** *P*<0.0001, NS no significance.

**Supplementary Figure S3**

**a**-**b** Combination treatment with JQ1 (10 μM) and the ferroptosis inhibitor fer-1 (1 μM) enhanced the invasion (**a**) and migration (**b**) abilities more noticeably than JQ1 alone in the MDA-MB-231 and Hs578T BRCA cell lines and in the A549 LUAD cell line. **c**-**d**: Cell counts in the invasion (**c**) and migration (**d**) assays. **e**-**f** Combination treatment with JQ1 (10 μM) and the ferroptosis inducer RSL3 (1 μM) inhibited invasion (**e**) and migration (**f**) more appreciably than either agent alone in the MDA-MB-231 and Hs578T BRCA cell lines and in the A549 LUAD cell line. **g**-**h** Cell counts in the invasion (**g**) and migration (**h**) assays. An equal volume of DMSO was used as the control. Scale bar =65 μm. * *P*<0.05, ** *P*<0.01, *** *P*<0.001. The data represent the results of at least three independent experiments.

**Supplementary Figure S4**

**a** The ratio of siRNA targeting ATG5 and ATG7. **b**-**c** The level of iron (**b**) and ROS (**c**) was increased under treatment with JQ1 (10 μM), and this increase was reversed under treatment with the combination of JQ1 (10 μM) and knockdown of ATG5 in the MDA-MB-231 and Hs578T BRCA cell lines and in the A549 LUAD cell line. **d-e** The level of iron (**d**) and ROS (**e**) was increased under treatment with JQ1 (10 μM), and this increase was reversed under treatment with the combination of JQ1 (10 μM) and knockdown of ATG7 in the MDA-MB-231 and Hs578T BRCA cell lines and in the A549 LUAD cell line. **f** Expression of ATG5, LAMP1 and S6K under treatment with JQ1 (10 μM, 24h). An equal volume of DMSO was used as the control, levels of iron and ROS were assessed after the application of the drugs for 24 h. The data represent the results of at least three independent experiments, * *P*<0.05, ** *P*<0.01, *** *P*<0.001. **g** The expression of BRD4 was negatively related to the expression of *ATG5* (R=-0.0373) and *LAMP1* (R=-0.3364) in pan-cancer patients (N=10323). The data were analyzed through the calculation of pairwise Pearson correlation coefficients.

**Supplementary Figure S5**

**a** The siRNA sequences targeting BRD4, ATG5 and ATG7. **b** The sequence of the recombinant plasmid expressing GPX4. **c** The sequences of the primers used in Real-time PCR.
